# The Neuroproteomic Basis of Enhanced Perception and Processing of Brood Signals That Trigger Increased Reproductive Investment in Honeybee (*Apis mellifera*) Workers

**DOI:** 10.1074/mcp.RA120.002123

**Published:** 2020-11-25

**Authors:** Xufeng Zhang, Han Hu, Bin Han, Qiaohong Wei, Lifeng Meng, Fan Wu, Yu Fang, Mao Feng, Chuan Ma, Olav Rueppell, Jianke Li

**Affiliations:** 1 Institute of Apicultural Research/Key Laboratory of Pollinating Insect Biology, Ministry of Agriculture, Chinese Academy of Agricultural Sciences, Beijing, China; 2 Institute of Horticultural Research, Shanxi Academy of Agricultural Sciences, Shanxi Agricultural University, Taiyuan, China; 3 Department of Biology, University of North Carolina at Greensboro, Greensboro, North Carolina, USA

**Keywords:** Label-free quantification, neurobiology, tissues, mass spectrometry, quantification, antennal lobes, Apis mellifera, brain proteome, mushroom bodies, reproductive investment

## Abstract

The neuronal basis of complex social behavior is still poorly understood. In honeybees, reproductive investment decisions are made at the colony-level. Queens develop from female-destined larvae that receive alloparental care from nurse bees in the form of ad-libitum royal jelly (RJ) secretions. Typically, the number of raised new queens is limited but genetic breeding of “royal jelly bees” (RJBs) for enhanced RJ production over decades has led to a dramatic increase of reproductive investment in queens. Here, we compare RJBs to unselected Italian bees (ITBs) to investigate how their cognitive processing of larval signals in the mushroom bodies (MBs) and antennal lobes (ALs) may contribute to their behavioral differences. A cross-fostering experiment confirms that the RJB syndrome is mainly due to a shift in nurse bee alloparental care behavior. Using olfactory conditioning of the proboscis extension reflex, we show that the RJB nurses spontaneously respond more often to larval odors compared with ITB nurses but their subsequent learning occurs at similar rates. These phenotypic findings are corroborated by our demonstration that the proteome of the brain, particularly of the ALs differs between RJBs and ITBs. Notably, in the ALs of RJB newly emerged bees and nurses compared with ITBs, processes of energy and nutrient metabolism, signal transduction are up-regulated, priming the ALs for receiving and processing the brood signals from the antennae. Moreover, highly abundant major royal jelly proteins and hexamerins in RJBs compared with ITBs during early life when the nervous system still develops suggest crucial new neurobiological roles for these well-characterized proteins. Altogether, our findings reveal that RJBs have evolved a strong olfactory response to larvae, enabled by numerous neurophysiological adaptations that increase the nurse bees' alloparental care behavior.

## Teaser

We explore the basis for one of the fundamental tradeoffs in life: to invest or not to invest in reproduction of queen bee. We demonstrate measurable differences in the behavior of high royal jelly-producing bees (RJBs) vs “normal” honeybees. The RJBs evolve a strong olfactory response to larvae, neurophysiological adaptations by inducing processes of energy and nutrient metabolism, signal transduction that increase the nurse bees' alloparental care behavior.

## Graphical Abstract

**Figure:**
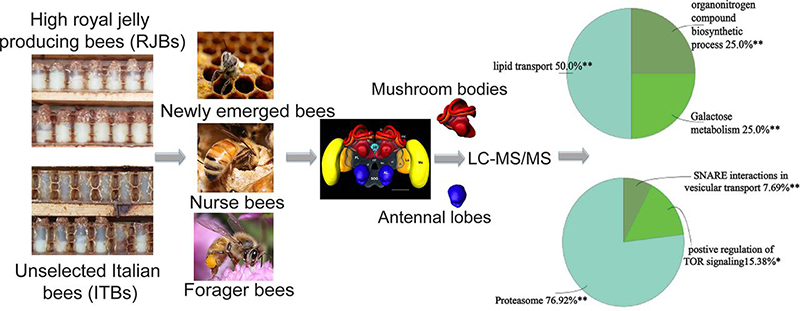

**Highlights**

• The RJB syndrome is mainly due to a shift in nurse bee alloparental care behavior.
• The RJB nurses spontaneously respond more often to larval odors compared to ITB nurses but their subsequent learning ability are similar.
• The RJBs evolve neurophysiological adaptations that increase the nurse bees' alloparental care behavior.
• Major royal jelly proteins and hexamerins play crucial neurobiological roles for the nervous system.

Honeybees are important model organisms for studying complex social behavior, such as communication, division of labor, and learning and memory in the context of the colony (1, 2, 3, 4). Alloparental care of the young brood by nurse bees is critical for growth and reproduction of honeybee colonies. The care involves feeding larvae with royal jelly (RJ) secreted from the nurses' hypopharyngeal, mandibular, postcerebral, and thoracic glands (5, 6, 7). The perception of active brood signals causes nurse bees to provide the growing larvae with food (8). However, the neurobiological processing of brood signals and nursing in general is little understood in honeybees, even though it is likely that the behavior is ultimately regulated by central, higher order processes in the brain (9).

The honeybee brain has distinct anatomical subdivisions to execute different functions (10). The paired mushroom bodies (MBs) contain >300,000 neurons and integrate sensory information, whereas the optic lobes (OLs) and antennal lobes (ALs) are the primary sensory centers which send their projections to the MBs (3, 10, 11, 12). The ALs contain 165 spherical neuropiles (glomeruli) that code different odors with their activity patterns, which is largely independent of olfactory learning (10). The size of the glomeruli can change with age and the behavioral profile of worker bees, presumably to accommodate the different demands for processing information (13). The MBs and ALs of the honeybees are the primary constituents of olfactory processing and connect via a combinatorial network (14). MB-extrinsic neurons of honeybees display strong neural plasticity in the formation of olfactory memory (15, 16, 17). However, the complementary roles of MBs and ALs in complex social behavior, such as nursing and reproductive investment decisions, have yet to be fully studied.

A few complex social behaviors of honeybees have been correlated with transcriptomic changes in the brain, demonstrating that numerous physiological changes underlie the behavioral plasticity of honeybees. For example, the transition from in-hive activities to foraging has been characterized in detail at the transcriptome level of the entire brain (18, 19). At the anatomical level, specific changes in the volume of the MBs have been documented during this transition (20), but connections between the transcriptome or proteome of specific brain structures and complex behavioral phenotypes are still rare. A few notable exceptions include the transcriptomic characterization of the mushroom bodies in response to short-term social stress (21), developmental changes during the transition from young in-hive workers to foragers (22), and a combination of short- and long-term behavioral changes (23). Furthermore, species differences in specific brain structures have been correlated with behavioral differences between the Western honeybee, *Apis mellifera*, and the Eastern honeybee, *A. cerana* (24).

Within *A. mellifera*, artificial selection for commercial RJ production has generated pronounced increases in female reproductive investment. This selected stock of royal jelly bees (RJBs) raises many more new queens and provides each cell with more RJ than the stock of Italian bees (ITBs) (25, 26). This behavioral change is partly due to an increased responsiveness of the nurse bee antennae to larval pheromones (27). However, whole-brain studies also showed differences in neuropeptides, proteome, and phosphoproteome between the RJBs and ITBs (28, 29), indicating that central processing of the larval signals also differs between RJBs and ITBs. We present here a more detailed comparative study of the neuronal underpinnings of the different nursing behavior of RJBs and ITBs. Firstly, we confirm that their phenotypic differences in queen rearing are indeed due to the nurses. Then, we show with olfactory conditioning of the proboscis extension reflex that the spontaneous responsiveness to larval pheromones is increased in RJB compared with ITB nurse bees. Finally, we corroborate these results with comparative proteome characterizations of the ALs and MBs, showing that differences between the RJBs and the ITBs across three life stages predominantly occur in the ALs but not the MBs.

## EXPERIMENTAL PROCEDURES

##### Chemical Reagents

All chemicals were purchased from Sigma-Aldrich (St. Louis., MO, USA), and modified sequencing-grade trypsin was bought from Promega (Madison, WI, USA). All the chemical reagents were of analytical grade or HPLC grade.

##### Experimental Design and Statistical Rationale

The colonies used for collecting samples were raised at the apiary of Institute of Apicultural Research, Chinese Academy of Agricultural Sciences, Bejing. The open mated queens of ITBs and RJBs were bought from commercial breeders in California (USA) and Pinghu City, Zhejiang province of China, respectively. The queens were installed, and experiments started after the worker population had completely turned over.

**Fig. 1:**
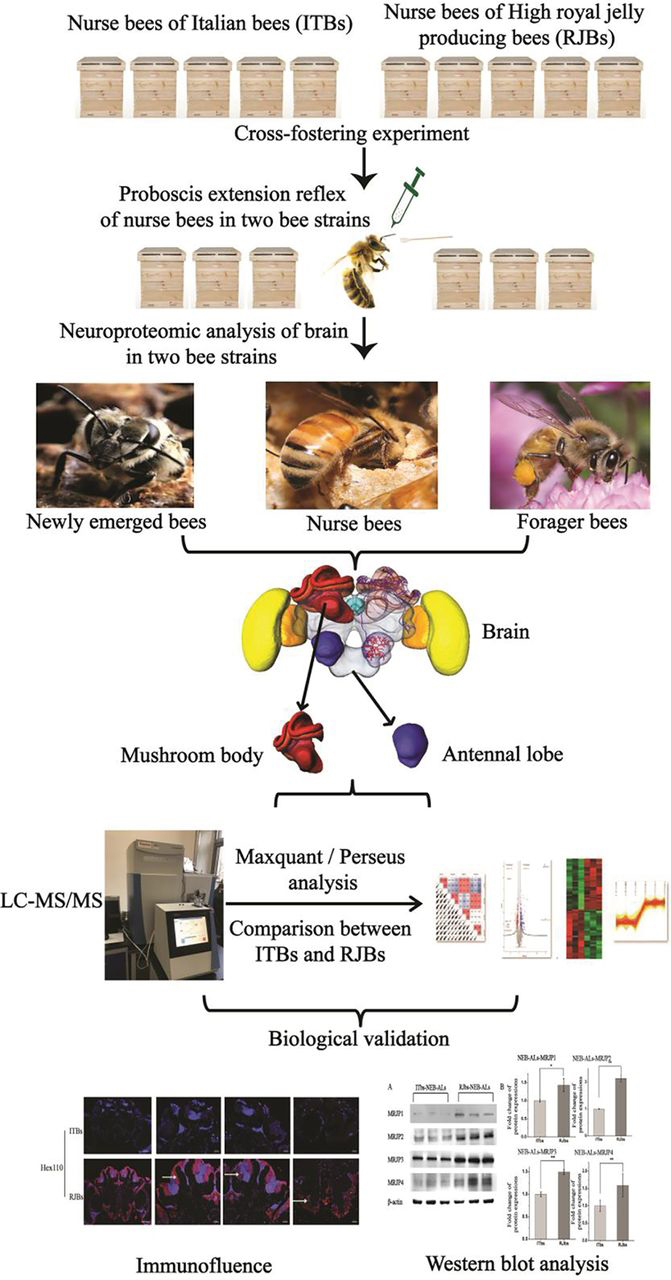
**The workflow chart represents experimental procedure.** Honeybee photos are provided by Professor Jianke Li. Brain photo is quoted from the book: Honeybee-Neurobiology-and-Behavior.

Experimental design and statistical rationale for each section of the whole experiments have been described separately, and workflow of the whole experiments is shown in Fig. 1. ten colonies (five colonies from each strain) were used for cross-fostering experiment, and then six colonies (three colonies from each bee stock) were used for olfactory conditioning of the proboscis extension reflex (PER) experiment. After PER experiment, the six colonies (three colonies from each strain) used in the PER experiment were collected for proteomic samples of subregion (mushroom bodies and antennal lobes) of bee brain in different stages. Honeybees from three colonies in each bee strain of different stages were collected and pooled as one sample respectively. For the different stages (at NEBs, NBs, FBs), at least 600 worker bees (∼200 worker bees/colony) were sampled in each bee strain of the different stages respectively. For LC–MS/MS analysis, technical triplicates were produced in each sample. Proteins were identified present only when they had a valid LFQ value (not NaN) in at least 2 replicates of each sample group. Pearson correlation, principle component analysis and volcano plots were done in Perseus. The biological validation of western blots was performed in biological and technical triplicates. The significant analysis of subregions of bee brain in different stage between two bee strains were counted by Perseus (Student's *t* test, S0 = 0.1, FDR = 0.05). In each bee strain, the validation of Immunofluorescence was performed by three brain samples.

##### Cross-Fostering Experiment

To compare the contributions of brood and colony environment to larval acceptance and RJ provisioning of larvae, ITB and RJB larvae were grafted into colonies of their own and the opposite stock (27). Five colonies of each stock with standardized adult population size, food stores, and brood pattern were used as source and recipient colonies for the experiment. Young worker larvae (∼24 h old) from a RJB or ITB colony were grafted into one frame containing 126 plastic queen cell cups fixed on two strips of wooden bars, which was then introduced into another colony of either RJB or ITB stock for nurse bees to take care of.

**Fig. 2:**
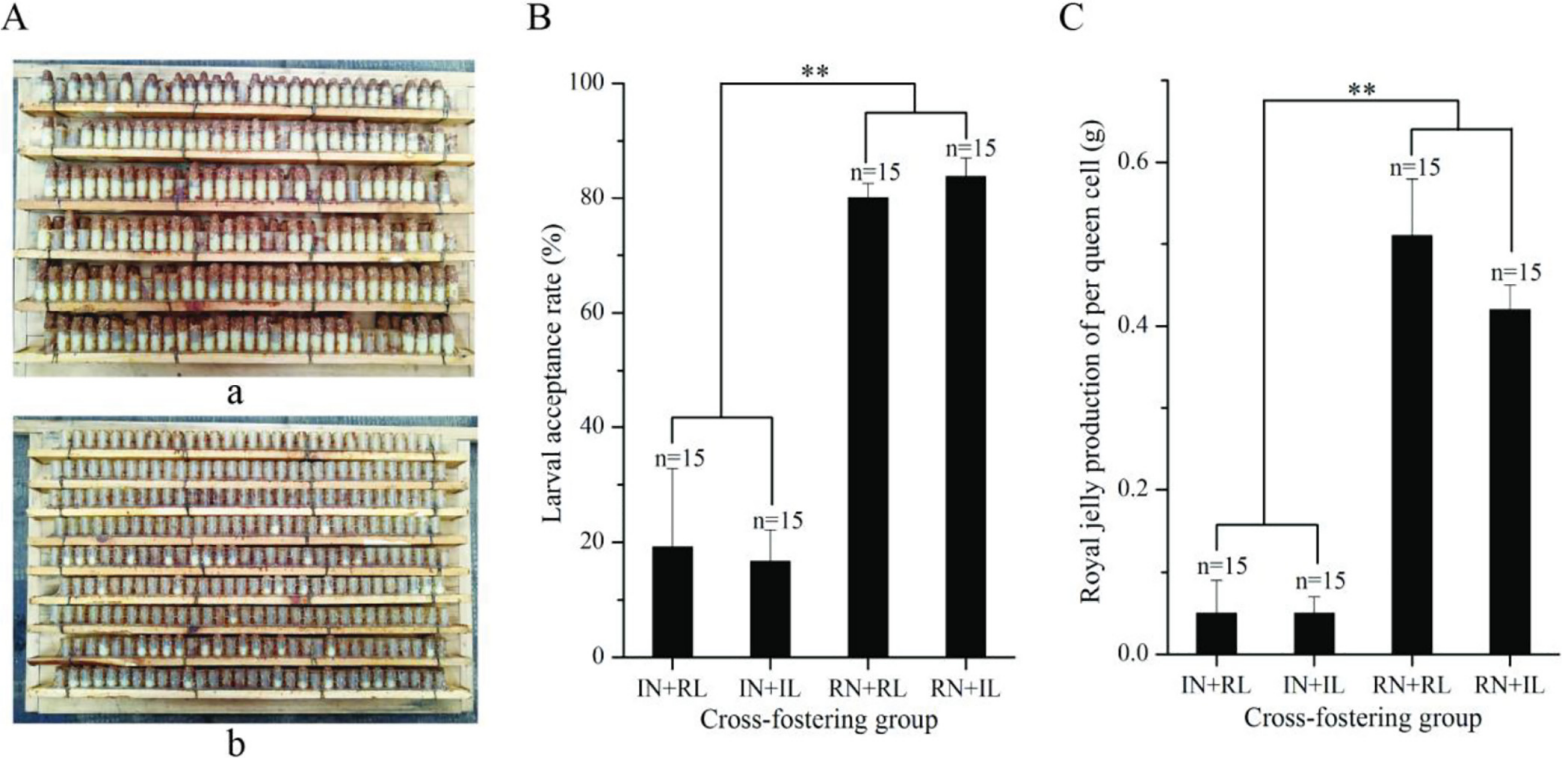
**Cross-fostering experiment between Italian bees (ITBs) and high RJ producing bees (RJBs) by grafting ITB and RJB larvae into colonies of the same or opposite stock.***A*, RJ in the queen cell cups of RJBs (panel a) and ITBs (panel b) 72h after larval grafting. *B*, Comparison of larval acceptance between ITB and RJB nurse bees with larvae of ITBs or RJBs (Mean±S.D., *n* = 15). *C*, Comparison of RJ production of per queen cell between ITB and RJB colonies with larvae of ITBs or RJBs (Mean±S.D., *n* = 15). “**” represents *p* < 0.01. IN: ITB nurse bees, RN: RJB nurse bees, IL: ITB larvae, RL: RJB larvae.

After 68∼70 h, the frames were taken out of the colony for RJ collection, according to standard practice (25, 30). All adult bees were removed from the frames and the acceptance of queen cells was estimated by counting the proportion of queen cells that contained larvae and RJ. The wax caps at the top of the plastic queen cell cups and the larvae in the cells were removed and the RJ was collected from all cells in a trial and weighed with an electronic scale (AL204-IC, Mettler Toledo, Switzerland). Measurement of larval acceptance and RJ production of each colony was performed with three different larval sources for 15 unique brood x nurse bee combinations in each experimental group (RJB x RJB, RJB x ITB, ITB x RJB, ITB x ITB) (Fig. 2). Acceptance was compared with separate, pairwise Fisher's exact tests across all replicates of two experimental groups, and RJ amounts were compared with a 2-factorial ANOVA, followed by post-hoc testing of individual effects. All the ITBs and RJBs were maintained at the apiary of the Institute of Apicultural Research, at the Chinese Academy of Agricultural Sciences in Beijing.

##### Olfactory Conditioning of the Proboscis Extension Reflex Experiment

**Fig. 3:**
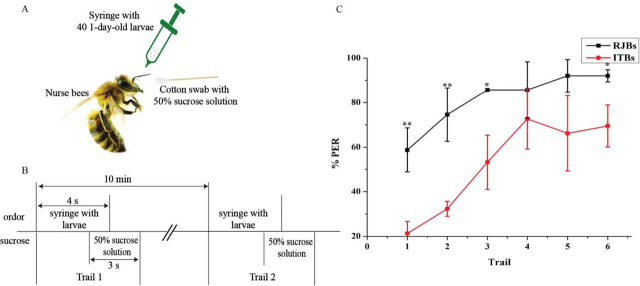
**Proboscis extension response (PER) of nurse bees of two honey bee strains (high royal jelly producing bees, RJBs) and Italian bees (ITBs) in response to brood odors. RJB and ITB nurses with differ in reproductive investment and this experiment showed a major difference in initial response and a minor difference in learning between the two bee stocks.***A* and *B*, show the experimental procedure of olfactory PER conditioning with larval odors. *C*, Percentage of individuals exhibiting conditioned PER to larval odor cues differed significantly between the nurse bees of ITBs and RJBs (Mean±S.D., “**” represents *p* < 0.01, “*” represents *p* < 0.05, *n* = 63 nurse bees for three replicates in total per stock). Spontaneous PER after stimulation with larval odors (trial 1) was significantly higher in RJBs than in ITBs, explaining all subsequent differences.

To test for differences between RJB and ITB nurse bees in responsiveness to and learning of larval odors, individual workers were trained to associate larval odors with a sucrose reward in the olfactory conditioning of the proboscis extension reflex (PER) (Fig. 3). Individuals from three standardized colonies of each stock were used. The PER set-up and training routine followed previously described protocols (31). Nurse bees, identified by their feeding of larvae in queen cells, were collected from the colonies and directly anesthetized with CO_2_ for a few seconds. Before they awoke, the bees were placed into a plastic straw with a diameter of 6 mm and a pin was used to fix them between the thorax and abdomen to prevent movements of their body parts, except their mouthparts and antennae. Twenty-one bees of each colony were collected and tested for a total sample size of 63 bees per stock.

Harnessed bees were fed with a drop of 50% sucrose solution before the experiment. Bees that did not perform the PER when receiving 50% sucrose solution were discarded. Likewise, harnessed bees that spontaneously responded to a neutral air puff delivered by an empty syringe to the antennae for 4s were excluded. All excluded individuals were replaced to maintain a sample size of 21 per colony. Sucrose response thresholds are important in PER conditioning (32) but potential differences between ITBs and RJBs were considered meaningful and therefore not controlled for. Then, another syringe containing 40 one-day-old living larvae from unrelated colonies was used to blow onto the antennae of the harnessed bees for 4s, followed by a stimulation of 50% sucrose solution with 1s overlap. The harnessed bees were conditioned for six trials of paired brood odor-sucrose presentations with an average inter-trial interval of 10 min between trials. Three variables were compared between ITBs and RJBs across colonies with Mann-Whitney U-tests: the number of learning trials until the first positive PER response, the total number of positive PER responses, and the number of nonresponses after the first correct response. In addition, Fisher's exact tests were used to compare ITBs and RJBs with respect to the proportion of bees that spontaneously responded to larval odors among all bees tested and the proportion of learners among all bees that did not spontaneously respond.

##### Comparative Proteomic Analysis of Antennal Lobes and Mushroom Bodies

RJB and ITB combs with mature pupae were placed into an incubator (34 °C and 80% relative humidity) overnight to collect newly emerged worker bees. Older nurse bees were identified by feeding behavior of larvae and collected directly from the brood nest. Forager bees were identified by returning to the hive with pollen loads on their hind legs and they were collected at the hive entrance. For newly emerged bees, nurses, and foragers of each bee strain ∼600 worker bees (∼200 worker bees/colony) were sampled. The sampled bees were anesthetized with CO_2_ for a few seconds to allow accurate dissection. Mushroom bodies (MBs) and antennal lobes (ALs) were dissected from the head capsule according to previously described methods (33), adding cold protease inhibitor mixture. The dissected samples were immediately pooled and stored at −80 °C until further analysis.

##### Protein Extraction, Digestion and LC–MS/MS Analysis

Total protein was extracted from the frozen MBs and ALs (∼200 worker bees per each of 3 colonies of the RJBs and ITBs) respectively as described previously (24). Proteins from the tissues of bee brain were extracted by using acetone precipitation method. Protein samples were digested with sequencing grade trypsin in a specific volume ratio (enzyme: protein = 1:50) at 37 °C overnight. The digest was stopped by adding 1μl of formic acid. The digested peptides were centrifuged at 12,000 × *g* for 10 min at room temperature (RT) and were desalted using C18 columns (Thermo Fisher Scientific, Bremen, Germany). The peptide samples were dried in a SpeedVac system (RVC 2-18, Marin Christ, Osterod, Germany) and stored at −80 °C for subsequent LC–MS/MS analysis.

The digested peptide samples were dissolved in 0.1% formic acid and analyzed using an EASY-nLC 1200 (Thermo Fisher Scientific, Bremen, Germany) coupled with Q Exactive HF (Thermo Fisher Scientific, Bremen, Germany) via an ESI ion source (Thermo Fisher Scientific). First, peptides were loaded with a trap column (2 cm long, 100 μm inner diameter fused silica filling with 5.0 μm Aqua C18 beads, Thermo Fisher Scientific) in buffer A (0.1% formic acid in water) for 2 min at a flow rate of 5 μl/min. Secondly, peptides were separated on an analytical column packed with 3 μm, 100 Å, Aqua C18 beads (15 cm long, 75 μm inner diameter, Thermo Fisher Scientific) at a flow rate of 350 nL/min. A 120 min gradient was performed to elute the peptides: From 3% to 8% buffer B (0.1% formic acid in acetonitrile) in 5 min, from 8 to 20% buffer B in 80 min, from 20 to 30% buffer B in 20 min, from 30 to 90% buffer B in 5 min, and remaining at 90% buffer B for 10 min.

The peptides were injected into a Q Exactive HF mass spectrometer (Thermo Fisher Scientific) through electrospray ionization. MS and MS/MS data were collected in data-dependent acquisition mode and full scans were collected with the following settings: resolution at 120,000; scanning range from *m*/*z* 350-1550 at *m*/*z* 400; AGC target: 3 × 10^6^; MIT: 20 ms. Top 20 MS/MS scans were collected by using higher-energy collision induced dissociation (HCD) in the linear ion trap mass spectrometer with the following settings: Resolution at 15000; AGC target: 1 × 10^5^; maximum inject time (MIT): 35 ms; isolation window: 2.0 *m*/*z*; normalized collision energy: 28; loop count 20; dynamic exclusion with a repeated count: 1; exclusion duration: 40s; charge exclusion: unassigned 1, 7, 8, >8; peptide match: preferred; exclude isotopes: On. The MS/MS spectra were retrieved by using Xcalibur (version 4.0, Thermo Fisher Scientific). The MS proteomics data have been deposited to the ProteomeXchange Consortium (http://proteomecentral.proteomexchange.org) via the iProX partner repository (34) with the data set identifier PXD014207.

##### Protein Identification and Label-Free Quantification of Protein Abundance

The MS/MS raw data were processed with the software MaxQuant (version 1.6.1.0) (35) and the Andromeda search engine (36). Proteins were identified by searching against the database sequence of *Apis mellifera* (22460 protein sequences, downloaded on 18 April, 2017 from the NCBI database) together with a list of common contaminants. MaxQuant search settings were: fixed modification, Carbamidomethylation(C); variable modifications, acetylation (protein N-term); maximum number of modifications per peptide of 5; maximal missed cleavages of 2; the first search peptide: 20 ppm, main search peptide: 4.5 ppm; the MS/MS match tolerance: 20 ppm; Enzyme, Trypsin/P. Relative protein quantification was performed using the LFQ algorithm of MaxQuant (37). Proteins were identified based on at least one unique peptide with a label minimum ratio count of one, unique and razor peptides for quantification; maximum false peptide and protein discovery rates of 0.01; for matching between runs, 0.7 min match time window, and 20 min alignment time window.

##### Bioinformatics Analysis

The protein lists generated by MaxQuant were imported and further analyzed by using the Perseus software (version 1.6.1.1, http://www.coxdocs.org/doku.php?id=perseus:start/). Proteins which were matched to the reverse (or contaminants) database, or identified only by modified peptides, were filtered out. In a second filtering step, proteins were excluded when they did not have at least valid values for two or more label-free quantification (LFQ) values in at least one study group. LFQ values were then transformed in log_2_X. The proteins were statistically analyzed using t-tests, using FDR = 0.05, S0 = 0.1, and 250 as randomization number on the Perseus platform. Euclidean distance was used for hierarchical clustering, and principle component analysis was performed after imputation with the following parameters: width 0.3, down shift 1.8, and total matrix mode. The protein lists were characterized by functional gene ontology analyses of the biological processes and Kyoto Encyclopedia of Genes and Genomes (KEGG) pathway analyses with ClueGO (version 2.5.1) and the Cytoscape plug-in (http://www.ici.upmc.fr/cluego/) software (version 3.6.1) (38). These enrichment analyses were performed by comparing an input data set of identified proteins to all functionally annotated GO categories in the entire genome of *Apis mellifera* from the NCBI database. The significantly enriched GO terms and KEGG pathways were reported using a two-sided hyper geometric test and only *p* ≤ 0.05 were considered after Benjamini-Hochberg correction that corresponded to false discovery rate of 0.05. Functional grouping of the terms was based on GO hierarchy. The tree level was varied from 3 to 8, and kappa score level was 0.4. For comparison purposes, terms were merged when 60% of the genes were shared.

##### Verification of Protein Differences with Western Blotting and Immunofluorescence

To further verify select candidate proteins that were differentially abundant between RJB and ITB stocks, proteins associated with energy synthesis and metabolism in newly emerged bees, and proteins related to signal-transduction pathways in nurse bees were studied. These candidates were vitellogenin (Vg), larval-specific very high density lipoprotein (VHDL), major royal jelly protein 1 (MRJP1), MRJP2, MRJP3, MRJP4, hexamerin 70a (Hex70a), hexamerin 70b (Hex70b), hexamerin 70c(Hex70c), hexamerin 110 (Hex110), syntaxin-17 (Syx17), tyrosine-protein kinase Src64B (Src64B), serine/threonine-protein kinase p21-activated kinase 3 (PAK3) and cyclic guanosine-3′, 5′-monophosphate (cGMP)-dependent protein kinase foraging (PKG). Polyclonal antibodies for Vg, VHDL, Hex70a, Hex70b, Hex70c, Syx17, PAK3, Src64B and PKG were developed in New Zealand female rabbits by Genecreat (Wuhan, China). While most of these antibodies were produced against the respective full-length proteins, Hex70a and Hex70b antibody production used only specific peptides. Monoclonal antibodies (mAbs) of MRJP1, MRJP2, MRJP3, and MRJP4 were developed in mice by Genecreat (Wuhan, China). A mAb of Hex110 was developed in mice by Abmart (Shanghai, China). The specificity of these antibodies was tested using ELISA (enzyme-linked immunosorbent assays) by Genecreat (Wuhan, China) and Abmart (Shanghai, China), respectively.

Equal amounts of protein (40 μg/lane) of 3 pooled samples were separated by stacking (5%) and separating (10%) SDS-PAGE gels, then transferred to a PVDF membrane (0.2 μm pore size) (Merck Millipore, USA) using an iBlot apparatus (Invitrogen). After blocking, the membrane was incubated overnight at 4 °C with primary antibodies at a dilution of 1:1000 (v/v). After washing 3 times with TBST, the membrane was incubated with the horseradish peroxidase-conjugated goat anti-rabbit or anti-mouse secondary antibody in a dilution of 1:5000 (v/v) for 1h. Antibody reactive bands were visualized using the ECL western blotting substrate (Pierce, Rockford, IL) and the binding reaction was quantified by densitometry using photographic film exposure. β-actin was detected simultaneously as an internal control. The ImageJ software package was used to calculate the intensity of the bands.

Immunofluorescence was used to compare the protein abundance of MBs and ALs *in situ* between the ITB and RJB stocks. The same candidate genes, related to energy synthesis and metabolism in newly emerged bees and signal transduction in nurse bees, were tested. Newly emerged bees and nurse bees (30 RJBs and 30 ITBs) were anesthetized with CO_2_ for a few seconds before their brains were dissected in cold 6.7 mM phosphate-buffer saline (PBS) with a protease inhibitor mixture. The whole intact brain was fixed in 4% paraformaldehyde at room temperature. Samples were dehydrated by ethanol with increasing grades from 50% to 100%, followed by xylene, and finally incubated and embedded in paraplast for sectioning. The slices were rehydrated in ethanol by decreasing grades from 100% to 50%, followed by three washes with 0.01M PBS and transfer to a 3% hydrogen peroxide solution for incubation for 10 min in the dark.

Afterward, the slices were washed 3 times for 5 min with 0.01M PBS, and then transferred to hydrogen peroxide solution (3%) and incubated for 10 min in the dark. Subsequently, the slides were transferred to BSA (5%) for 20 min at RT to block nonspecific binding. The diluted primary antibodies in BSA (Hex110 1:100) were applied to the slides and incubated overnight at 4 °C. Afterward, the slides were washed 3 times for 5 min in 0.01M PBS and incubated with anti-mouse or anti-rabbit IgG (diluted 1:50 in blocking solution) with Cy3 for 50 min at RT. Finally, the slides were washed 3 times for 5 min in 0.01M PBS, and covered with a drop of DAPI Fluoromount-GTM to reduce the amount of fluorochrome quenching while imaging with an OLYMPUS IX-51 inverted fluorescence microscope. Color intensities of the entire brain section on the slides were then measured in the Micro-Publisher 5 RTV (Q-imaging, CA) software.

## RESULTS

##### Cross-Fostering Experiment Identifies Effect of Host Colony but Not Brood

Reciprocal cross-fostering of larvae grafted into queen cells demonstrated a strong influence of the host colony and a minor, inconsistent effect of brood type (Fig. 2): In ITB colonies, the acceptance of queen cells containing ITB or RJB larvae was 16.30 ± 5.44% and 19.21 ± 13.65%, respectively. The rate for RJB brood was marginally higher (*n* = 3780, Fisher's exact *p* = 0.046; supplemental Table S1). In RJB colonies, the overall acceptance rate of queen cells was increased 4-5 times. Cells in RJB colonies containing ITB and RJB larvae were accepted in 83.81 ± 3.25% and 80.11 ± 2.53%, respectively, which indicated slightly higher acceptance of ITB brood (*n* = 3780, *p* = 0.004; supplemental Table S1). The larval acceptance in RJB colonies was significantly higher than those in ITB colonies for RJB brood (*n* = 3780, *p* < 0.001) and ITB brood (*n* = 3780, *p* < 0.001). Similarly, the biggest effect on RJ amount per cell was at the colony level (F_(1,56)_ = 115.8, *p* < 0.001) with much higher provisioning by RJB colonies (0.57 ± 0.12 g/cell) than ITBs (0.26 ± 0.13 g/cell). However, brood also had a small effect (F_(1,56)_ = 4.2, *p* = 0.044) and there was a significant interaction between both factors (F_(1,56)_ = 6.5, *p* = 0.014). Specifically, no significant effect of brood was found in ITB colonies (*p* = 0.765) but in RJB colonies, RJB brood received more RJ per cell (0.64 ± 0.11 g/cell) than ITB brood (0.51 ± 0.08 g/cell; *p* = 0.001).

##### Proboscis Extension Reflex Experiment Indicates a Stronger Response to Larval Pheromone in RJB than ITB Nurses

Spontaneous Proboscis Extension Response (PER) after stimulation with larval odors was significantly more common in RJBs than in ITBs (Fig. 3; *n* = 126, Fisher's exact *p* < 0.001). Correspondingly, the first response to larval odors across the six learning trials was also significantly earlier in RJBs than ITBs (Z = −5.2, *n* = 126, *p* < 0.001) and RJBs had a significantly higher overall PER score than ITBs across all trials (Z = 5.3, *n* = 126, *p* < 0.001). Within RJBs or ITBs, colonies were not significantly different for any of the evaluated variables. Excluding spontaneous responders, a slightly higher proportion of RJB than ITB bees responded to the conditioned brood stimulus during trials 2–6 (*n* = 76, Fisher's exact *p* = 0.040).

##### Overall Patterns of Proteomics Data

Correlations between ITBs and RJBs were high (R_S_ = 0.97–0.99; supplemental Fig. S1) for samples from the same brain region and behavioral state (newly emerged, nurse, forager). Among differing brain regions and behavioral states, the proteomes were also similar but not as highly correlated (R_S_ = 0.82–0.92; supplemental Fig. S1). Proteome profiles were overall grouped into the following four clusters: MBs of ITB and RJB nurse and foragers bees, ALs of ITB and RJB nurse and forager bees, ALs of ITB and RJB newly emerged bees, and MBs of ITB and RJB newly emerged bees (supplemental Fig. S2A). Small differences between ITBs and RJBs were apparent in the principal component analysis for ALs and MBs of all life history stages, although the separation was more distinct in the ALs (supplemental Fig. S2B).

##### Proteome Comparisons

Numerous protein groups (2495-2868) were identified from both the ALs and MBs of ITBs and RJBs in each age group (Table I). About 1% of these protein groups in each experimental comparison between ITBs and RJBs were uniquely represented in one or the other bee strain. On average, a slightly larger fraction of protein groups exhibited quantitative differences between ITBs and RJBs in the corresponding comparisons (Table I).

**Table I:** Summary of the proteomic comparisons of antennal lobes (ALs) and mushroom body (MBs) between high royal jelly producing bees (RJBs) and unselected Italian bees (ITBs) in newly emerged bees (NEB), nursing bees (NB), and forager bees (FB) stage

Contrast	# of Unique/Total Protein Groups in RJBs	# of Unique/Total Protein Groups in ITBs	Up-Regulated in RJBs		Up-Regulated in ITBs	
			# of Protein Groups	GO Terms	# of Protein Groups	GO Terms
NEB–ALs	31/2782 (Fig. 4*A*, supplemental Table S2)	34/2785 (Fig. 4*A*, supplemental Table S2)	97 (supplemental Table S3)	lipid transport, organonitrogen compound biosynthetic process, Galactose metabolism (Fig. 4*B*&4*C*, supplemental Table S4)	80 (supplemental Table S3)	intracellular protein transport, cellular homeostasis, protein processing in endoplasmic reticulum functions (supplemental Fig. S3, supplemental Table S4)
NEB–MBs	21/2850 (Fig. 6*A*, supplemental Table S5)	39/2868 (Fig. 6*A*, supplemental Table S5)	38 (supplemental Table S6)	organic substance transport (Fig. 6*B*, supplemental Table S7)	22 (supplemental Table S6)	—
NB–ALs	44/2521 (Fig. 7*A*, supplemental Table S8)	18/2495 (Fig. 7*A*, supplemental Table S8)	115 (supplemental Table S9)	positive regulation of TOR signaling, proteasome, and SNARE interactions in vesicular transport (Fig. 7*B*, supplemental Table S10)	159 (supplemental Table S9)	mRNA transport, mRNA metabolic process and splicing, splicesome (supplemental Fig. S5, supplemental Table S10)
NB–MBs	21/2723 (supplemental Fig. S4A, supplemental Table S11)	16/2718 (supplemental Fig. S4A, supplemental Table S11)	34 (supplemental Table S12)	glycine, serine and threonine metabolism, nucleoside monophosphate metabolic process related to signal transduction (supplemental Fig. S6, supplemental Table S13)	16 (supplemental Table S12)	neuroactive ligand-receptor interaction pathways (supplemental Table S13)
FB–ALs	58/2673 (supplemental Fig. S4B, supplemental Table S14)	65/2680 (supplemental Fig. S4B, supplemental Table S14)	122 (supplemental Table S15)	translation, peptide and amide biosynthetic processes, and ribosome (supplemental Fig. S7, supplemental Table S16)	349 (supplemental Table S15)	—
FB–MBs	26/2588 (supplemental Fig. S4C, supplemental Table S17)	11/2573 (supplemental Fig. S4C, supplemental Table S17)	4 (supplemental Table S18)	—	6 (supplemental Table S18)	—

**Fig. 4:**
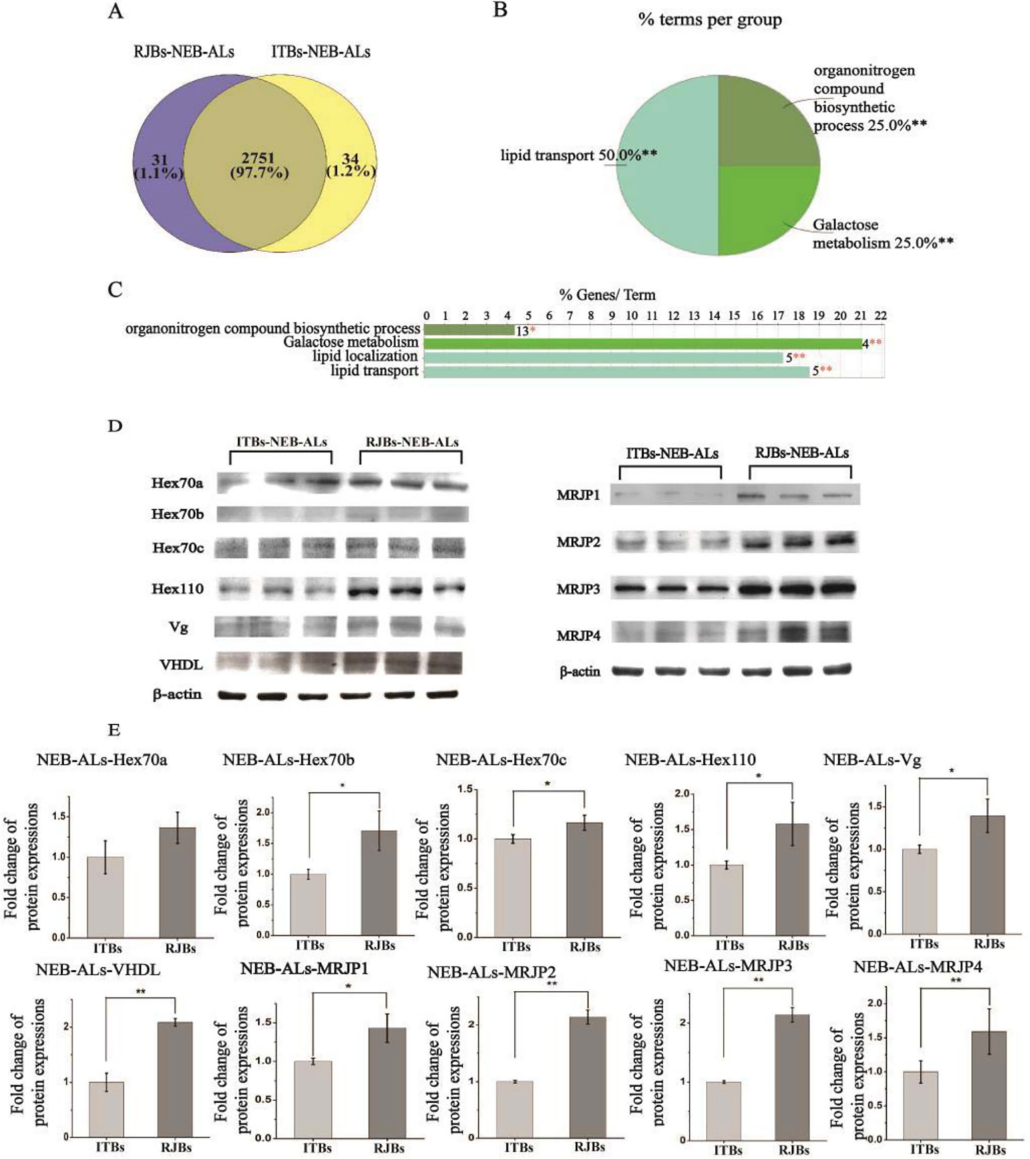
**Comparison of the proteome of antennal lobes (ALs) between newly emerged bees (NEB) from unselected Italian (ITBs, 3 pools each including 200 individual samples) and high royal jelly producing (RJBs, 3 pools each including 200 individual samples) honey bee stocks.***A*, Venn diagram performing the shared and unique protein groups identified in ITBs and RJBs (>97% are shared). *B* and *C*, The enriched functional classes, and pathways in quantitatively up-regulated proteins in the ALs of RJBs relative to ITBs (S0 = 0.1, FDR = 0.05). The percentage of genes/term represents the proportion of genes enriched in the respective functional group. Identical color summarizes bars from the same functional group. For details of the enrichment analysis results, see supplemental Table S4. “*” represents *p* < 0.05; “**” represents *p* < 0.01. *D*, Confirmation of different abundance of candidate proteins in the ALs of newly emerged bees of ITBs and RJBs by western blots. β-actin is used as a control. *E*, Normalized fold changes of selected protein abundances in the ALs of newly emerged RJBs (*n* = 3) compared with ITBs (*n* = 3), tested by western-blots.

**Fig. 5:**
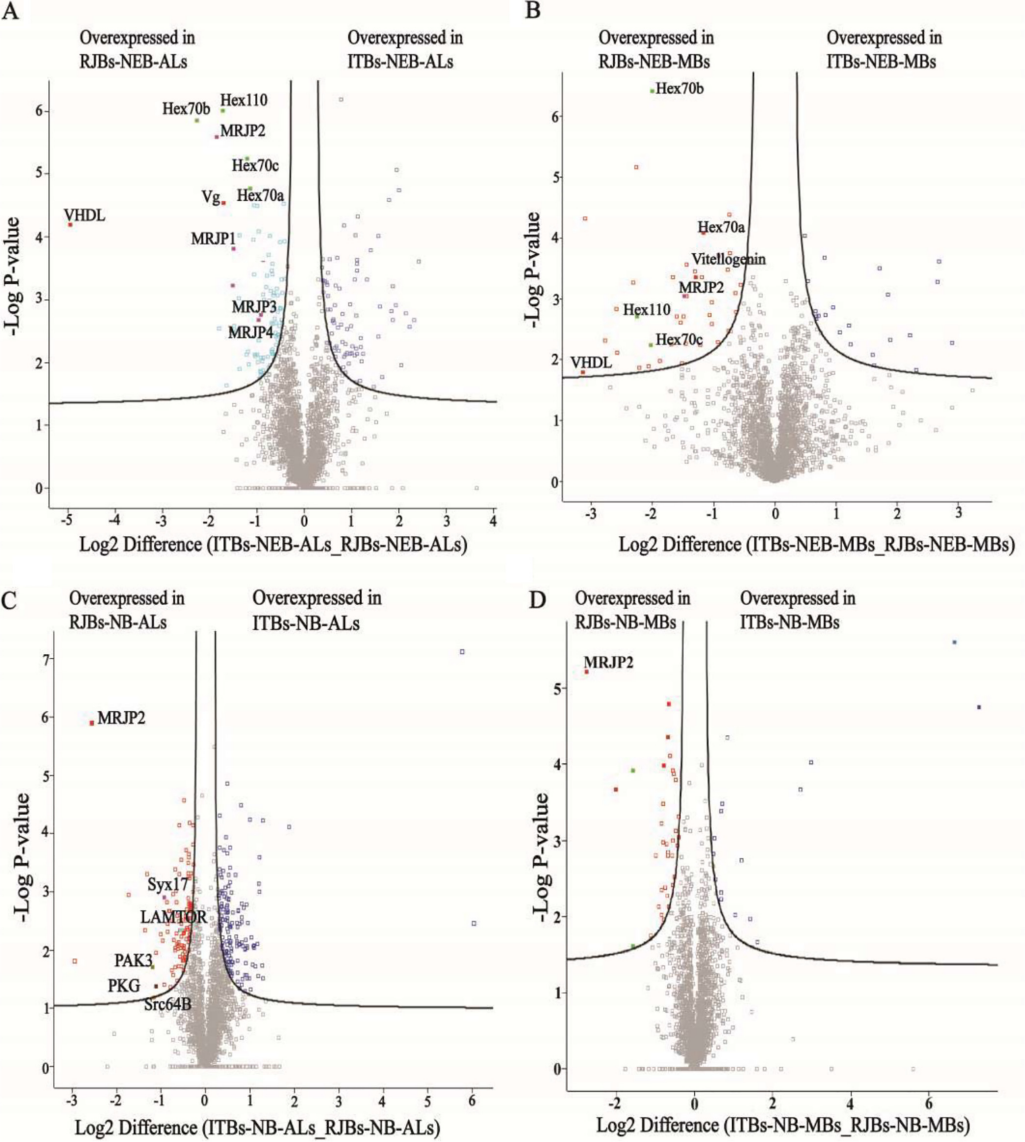
**Volcano plot of differentially abundant proteins. Differentially expressed proteins were obtained considering *p* < 0.05, S0 = 0.1.***A*, Volcano plot of differentially abundant proteins from the antennal lobes (ALs) comparison of unselected Italian bees (ITBs) and high royal jelly producing bees (RJBs) in newly emerged bees (NEB). *B*, Volcano plot of differentially abundant proteins from the mushroom bodies (MBs) comparison of ITB and RJB newly emerged bees. *C*, Volcano plot of differentially abundant proteins from the ALs comparison of ITB and RJB nurse bees (NB). *D*, Volcano plot of differentially abundant proteins from the MBs comparison of ITB and RJB nurse bees.

**Fig. 6:**
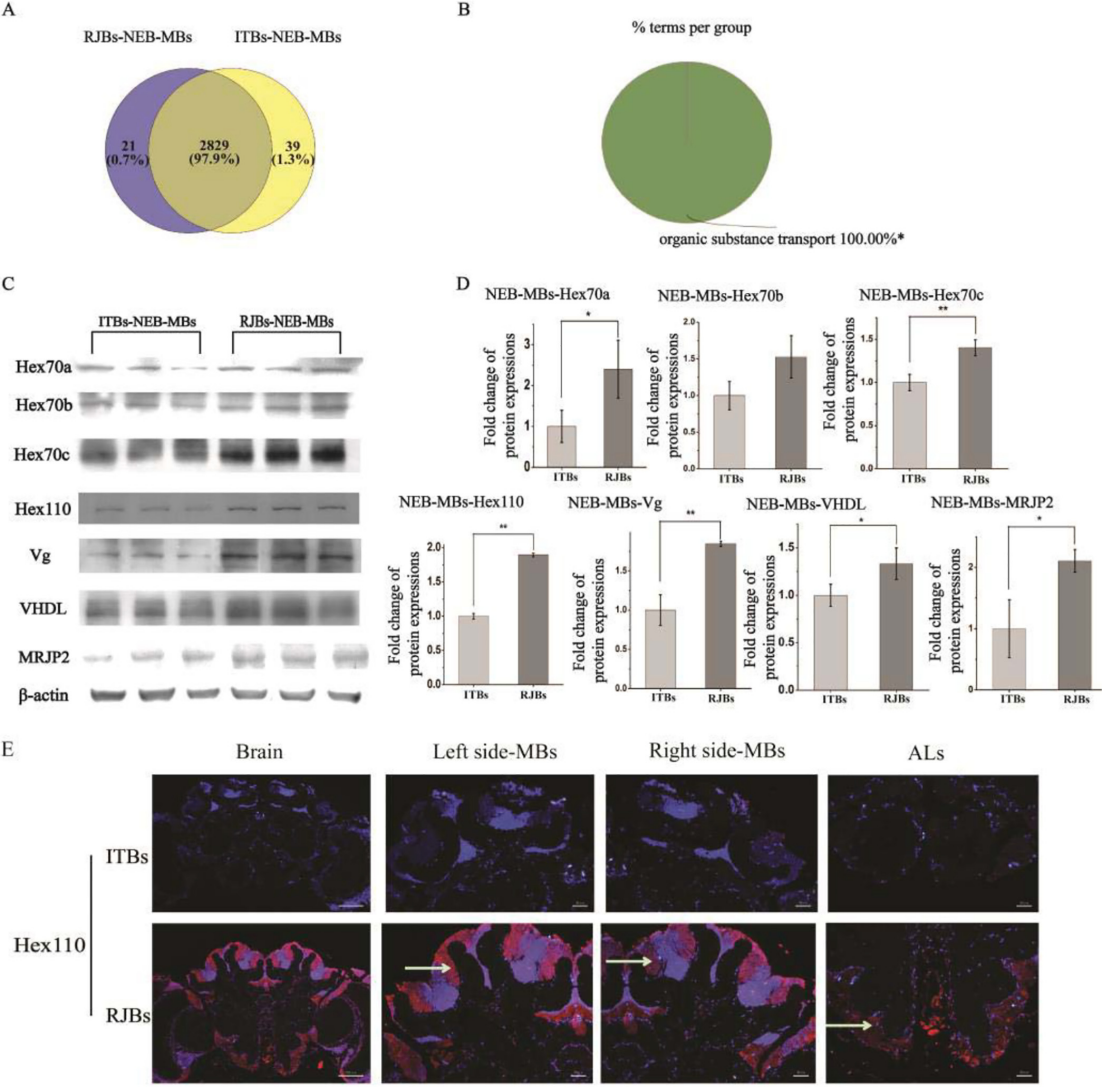
**Comparison of proteome of mushroom bodies (MBs) between newly emerged bees (NEB) from unselected Italian (ITBs) and high royal jelly producing (RJBs) honeybee stocks.***A*, Venn diagram representing the shared and unique protein groups identified in ITBs and RJBs (>97% are shared). *B*, The enriched functional classes and pathways of quantitative comparison by up-regulated proteins in the MBs of RJBs relative to ITBs (S0 = 0.1, FDR = 0.05). The percentage of genes/term represents the proportion of genes enriched in the respective functional group. Identical color summarizes bars from the same functional group. For details of the enrichment analysis results, see supplemental Table S7. “*” represents *p* < 0.05; “**” represents *p* < 0.01. *C*, Confirmation of different abundance of candidate proteins in the MBs of newly emerged bees of ITBs and RJBs by western blots. β-actin is used as a control. *D*, Normalized fold changes of selected protein abundances in the MBs of RJBs (*n* = 3) newly emerged bees compared with ITBs (*n* = 3), tested by western-blots. *E*, Immunostaining of brain sections with antibodies of Hex110 of newly emerged bees. Control staining with 4', 6-diamidino-2-phenylindole (DAPI). The red fluorescence represents the respective proteins, stained with Cy3-conjugated antibodies. Areas of particularly pronounced quantitative differences between RJB (*n* = 3) and ITB (*n* = 3) samples are indicated by white arrows. The scale bars for whole brain sections represent 200 μm; the scale bars of the MBs and ALs represent 50 μm.

In newly emerged bees, the up-regulated proteins in the ALs of RJBs compared with ITBs were enriched in “lipid transport,” “organonitrogen compound biosynthetic process,” and “galactose metabolism” (Fig. 4*B*). Prominent lipid transporters were the highly abundant vitellogenin (4 × up-regulated) and VHDL (32 × up-regulated) (Fig. 5*A*, supplemental Table S3 and S4). In addition, insect storage proteins Hex70a, Hex70b, Hex70c, Hex110 and the MRJPs (MRJP1, MRJP2, MRJP3, MRJP4) were all >3-fold more abundant in the ALs of RJBs than of ITBs (Fig. 5*A*, supplemental Table S3 and S4). Down-regulated proteins were enriched in “intracellular protein transport,” “cellular homeostasis,” and “protein processing in endoplasmic reticulum” (Fig. 4*B*, supplemental Fig. 3 and supplemental Table S4). Correspondingly, the MB proteome of RJBs was enriched in “organic substance transport,” including Vg and VHDL (Fig. 5*B* and 6*B*, supplemental Table S6 and S7), and some hexamerins and MRJP2 (Fig. 5*B*, supplemental Table S6), whereas proteins up-regulated in ITBs were not enriched in any GO term.

**Fig. 7:**
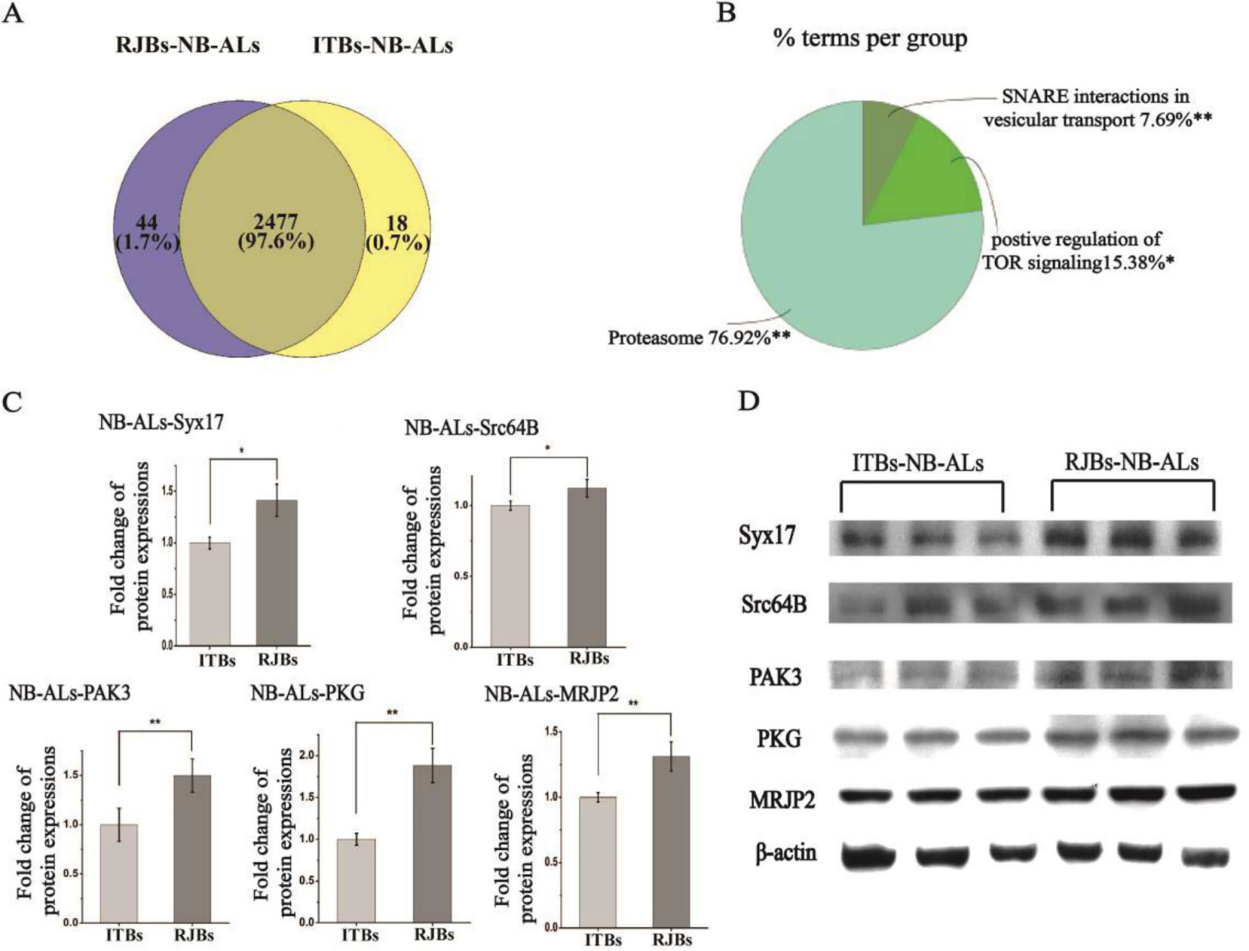
**Comparison of the proteome of antennal lobes (ALs) between nurse bees (NB) from unselected Italian** (ITBs, 3 pools each including 200 individual samples) and high royal jelly producing (RJBs, 3 pools each including 200 individual samples) honey bee stocks. *A*, Venn diagram representing the shared and unique protein groups identified in ITBs and RJBs (>97% are shared). *B*, The enriched functional classes and pathways of quantitative comparison by up-regulated proteins in the ALs of RJB nurse bees relative to ITBs (S0 = 0.1, FDR = 0.05). The percentage of genes/term represents the proportion of genes enriched in the respective functional group. Identical color summarizes bars from the same functional group. For details of the enrichment analysis results, see supplemental Table S10. “*” represents *p* < 0.05; “**” represents *p* < 0.01. *C*, Normalized fold change of selected protein abundance of the ALs of RJB nurse bees (*n* = 3) compared with ITBs (*n* = 3), based on all western-blot data. *D*, Confirmation of different abundance of candidate proteins in the ALs of nurse bees of ITBs and RJBs by western blots. β-actin is used as a control.

In nurse bees, differences were also more pronounced in the ALs than in the MBs. Proteins that were up-regulated in the RJBs compared with ITBs were enriched in “positive regulation of target of rapamycin (TOR) signaling,” “proteasome,” and “SNARE (soluble N-ethylmaleimide-sensitive factor attachment receptor) interactions in vesicular transport” (Fig. 7*B*, supplemental Table S9 and S10), whereas the most highly up-regulated proteins included MRJP2 and kinases Src64B, PAK3, and PKG (Fig. 5*C*, Fig. 5*D*, supplemental Table S9). Relative to the RJBs, the proteome of ITBs was enriched in seven pathways, most prominently “mRNA transport” and “mRNA metabolic processes and splicing” (supplemental Fig. S5 and supplemental Table S10). In the MBs of RJBs, up-regulated proteins were enriched in “glycine, serine and threonine metabolism” and “nucleoside monophosphate metabolic process related to signal transduction” (supplemental Fig. S6, supplemental Table S12 and S13), whereas down-regulated proteins were enriched in “neuroactive ligand-receptor interaction” (supplemental Table S12 and S13).

In foragers, numerous proteins were found in different quantities in the ALs of RJBs and ITBs but few quantitative differences between the ITBs and RJBs were found in the MBs. The up-regulated proteins in the ALs of RJBs were enriched “translation,” “peptide and amide biosynthetic processes,” and “ribosome” (supplemental Fig. S7, supplemental Table S15 and S16), whereas none of the other protein lists of the forager brains resulted in significant GO term enrichment.

##### Biological Validation

Quantitative differences between RJBs and ITBs for several candidate proteins (Fig. 5) were confirmed by Western blot analyses and immunofluorescence (Table II). Western blots verified that Vg, VHDL, Hex70a, Hex70b, Hex70c, Hex110, and MRJP2 in the MBs and ALs of newly emerged RJB bees were consistently more abundant than of ITBs (Fig. 4*D*, 4*E* and Fig. 6*C*, 6*D*). Moreover, the abundance of MRJP1, MRJP3 and MJRP4 in the ALs of newly emerged RJBs was higher than of ITBs (Fig. 4*D* and 4*E*). The immunofluorescence analysis confirmed the higher abundance of Hex110 in the MBs and ALs of RJBs compared with ITBs (Fig. 6*E*). In nurse bees, Syx17, Src64B, PAK3, PKG, and MRJP2 were confirmed by Western blot analysis to be more abundant in the ALs of RJBs than ITBs, and for MRJP2 also in the MBs (Fig. 7*C* and 7*D*).

**Table II:** Quantitative variation of selected proteins were validated by western blot and immunofluorescence in mushroom bodies (MBs) and/or antennal lobes (ALs)

Proteins'name	Samples	Proteomic Data	Western-Blots	ImmunoFluorescence
Vitellogenin	MBs, ALs in NEBs	√	√	
VHDL	MBs, ALs in NEBs	√	√	
He× 70a	MBs, ALs in NEBs	√	√	
He× 70b	MBs, ALs in NEBs	√	√	
He× 70c	MBs, ALs in NEBs	√	√	
Hex110	MBs, ALs in NEBs	√	√	√
MRJP1	ALs in NEBs	√	√	
MRJP2	MBs, ALs in NEBs and NBs	√	√	
MRJP3	ALs in NEBs	√	√	
MRJP4	ALs in NEBs	√	√	
Syx17	ALs in NBs	√	√	
Src64B	ALs in NBs	√	√	
PAK3	ALs in NBs	√	√	
PKG	ALs in NBs	√	√	

## DISCUSSION

The RJBs were selected from ITBs more than four decades ago and exhibit highly increased queen-rearing behavior, a social reproductive investment that requires the coordinated actions of many individuals. Identifying the intraspecific molecular variation in two central nervous tissues that underly the pronounced behavioral changes in this model system significantly advances our understanding of the neurobiological mechanisms that allow microevolution of complex social behavior, indicating predominantly regulatory changes in energy storage and metabolic pathways, including the honeybee-specific MRJPs.

Our behavioral findings confirm previous studies (27, 28, 29) that predominantly a higher responsiveness to brood odors by the nurse bees and not the larvae control the RJB phenotype. The RJBs exhibit an order or magnitude higher colony-level production of RJ (39) and the cross-fostering of RJB and ITB brood in RJB and ITB host colonies demonstrates that the vast majority of this effect is due to the host colony: Regardless of brood type, the RJB colonies accept 4-5 times more grafted queen cells and provision each cell with more than twice the amount of RJ. These data confirm previous findings (27), but our study also identifies minor brood effects. These effects are inconsistent between the two host colony environments, which indicates an interaction between the major differences in the nurses and some minor differences in the brood that previously might have remained undetected (27). Such interactions in social communication systems can be expected due to sender/receiver co-evolution (40).

The strong difference in spontaneous responders in our olfactory conditioning of the proboscis extension reflex corroborates the cross-fostering results. In contrast to the ITB nurses, a large fraction of RJB nurses responded spontaneously to the young brood odors in our PER experiment, suggesting that the RJBs are better at perceiving and responding to pheromones of young brood, such as beta- and allo-ocimene (27). The unconditioned PER provides valuable information about responsiveness that may be linked to sensory abilities (41) and we demonstrate here that it can occur in a context that is unrelated to food collection, although larval provisioning might have conceptual links to feeding behavior (42). To test learning and memory, spontaneous responders typically need to be excluded (43). After exclusion of the spontaneous responders, a small but significant difference in subsequent learning was observed, but the interpretation of this result is unclear. We cannot distinguish between an increased aptitude by the RJBs to learn brood odors or a higher evaluation of the sucrose reward, because we did not test for sucrose responsiveness. Regardless of its interpretation however, this effect is minor compared with the difference in spontaneous responsiveness.

The nurses' extension of the proboscis may be interpreted as a preparation for feeding behavior, but this has not been explicitly studied. When the PER test began, the RJB and ITB nurses had already been exposed to brood odors and nursing behavior. Therefore, they were not naive to the stimuli presented and it remains to be studied how much of the initial response difference is innate or due to previous exposure of RJBs and ITBs to queen-destined brood odors. In any case, these phenotypic results suggest adjustments in the antennal lobes (ALs) for processing the olfactory information to produce the RJB behavioral phenotype. The slightly higher subsequent learning performance of the conditioned brood odor stimulus in RJBs compared with ITBs indicated that some differences in the mushroom bodies (MBs), as the dominant neuropils for olfactory learning, could also be expected (14). These predicted molecular changes in response to RJB selection were reflected in the proteomes of the ALs and MBs at different life history stages and are discussed in more detail in the following section.

Our proteomic comparisons of two important brain regions between ITBs and RJBs newly identify specific molecular adaptations of the central nervous system that enable the RJB phenotype. In agreement with other studies (44, 45, 46), the results highlight the mechanistic importance of energy metabolism, transport, and storage for social behavior.

In newly emerged RJBs an increase in energy provisioning and metabolism was discovered compared with same-aged ITBs. The development of the complex central nervous system and neuronal activity of the brain requires a huge amount of energy (47, 48). The up-regulated proteins were related to galactose metabolism and lipid transport in the ALs and organic substance transport in the MBs of newly emerged RJBs, relative to ITBs. These quantitative changes could fuel brain development, new membrane formation, and neuropile expansion (49). The lipid transporter Vg and VHDL were also up-regulated in RJBs. Vg has a plethora of functions throughout the honeybee worker body (50, 51) and is found in glial cells of the honeybee brain (52) that aid the development and activity of neurons (53). VHDL, which was 32-fold and 9-fold more abundant in RJBs than ITBs in MBs and ALs, respectively, also plays a role in lipid transport and is related to vitellogenin (54, 55). All four distinct honeybee hexamerins, Hex70a, Hex70b, Hex70c and Hex110, were identified in the brain for the first time and exhibited a higher abundance in the newly emerged RJBs than their ITB counterparts. These proteins were abundant, suggesting vital roles of hexamerins in the brain in addition to their known functions during the post-metamorphic development in insects (56, 57).

RJBs secrete RJ on day 3 after emergence, much earlier than other honeybees (58). Thus, young RJBs mature faster to become nurses, which could be enabled by the stronger energy provision in the brain, specifically in the relevant ALs and MBs. The secretion of RJ is triggered by brood pheromone perception that is conveyed from the antenna to the ALs and MBs. These brood pheromones are more abundant in RJB colonies and better perceived by RJBs (27), which could lead to the observed proteome changes in the ALs and MBs (59). However, it is equally likely that the identified proteome differences are based on intrinsic genetic variation and common-garden experiments of individual workers will be required to distinguish these two alternative explanations. The proteome differences among ITB and RJB workers at this early developmental stage could occur to provide the energy required to process the perceived signals in the MBs and ALs of the RJBs, similar to the nurse bee stage (see below). However, we favor the idea that the up-regulated proteins are responsible for the faster development of the ALs and MBs, which is particularly likely for hexamerins and related storage proteins (60).

The artificial selection of RJBs has changed the antennal proteome of RJB nurse bees (27), and we predicted the strongest proteome changes in the ALs and MBs at this life history stage. Our data did not necessarily confirm this prediction in a quantitative or qualitative sense. However, numerous proteins were found in higher abundance in the nurse brains of RJBs than of ITBs, particularly in the ALs. Most notably, the target of rapamycin (TOR) is a nutrient-sensing Ser/Thr kinase that regulates cell growth and metabolism (61), and is also related to synaptic plasticity, learning and memory, and cognition (62). LAMTOR1, a member of the Ragulator/LAMTOR complex involved in mTOR signaling, has vital roles in regulating cell growth and energy homeostasis (63) and is highly abundant in the ALs of RJBs relative to ITBs. Three important kinases were also more abundant in the ALs of RJB nurses. Tyrosine protein kinase Src64B is a member of the Src family with potential importance for synaptic plasticity of the primary olfaction system in honeybees (24, 64). PAK3, a member of p21-activated kinases (PAKs) is pivotal for signal transduction (65). PKG, a serine/threonine kinase, takes part in neuronal processing of the olfactory system (66) and is implicated in learning, memory, and feeding behavior (67). Finally, the higher abundance of proteins involved in “SNARE interactions in vesicular transport,” such as syntaxin 17, in the ALs of RJB compared with the ITB nurses suggests that modulated vesicular transport and signal transduction may support the activity of neuronal cells. Moreover, syntaxin 17, localized in the endoplasmic reticulum (ER), plays a role in cell proliferation and differentiation (68, 69), which could be important for AL plasticity.

Larval feeding is initiated after the ALs process brood pheromone signals from the antennae and transmit then to the MBs via specific projection neurons (10). The MBs exhibited smaller proteome differences than the ALs, which corresponds to our behavioral data that the initial responsiveness, not learning, is mainly responsible for the nursing differences between RJBs and ITBs. We cannot exclude other unstudied brain structures to play an equally prominent role but the ALs and MBs are the prevalent olfactory processing centers (3), making the discussed proteins prime candidates for experimental follow-up studies. In contrast to our prediction, most quantitative and qualitative proteomic differences between RJBs and ITBs were found in the ALs at the forager stage. In contrast, the MBs of foragers exhibited few differences between RJBs and ITBs. None of these differences is likely to be responsible for the altered RJB nursing behavior. Instead, we interpret them as consequences of the individuals' previous life history, including a potentially different age of transitioning from nursing to foraging. This fundamental life history transition (70) has profound consequences for the proteome of the central nervous system in honeybees (71). Furthermore, the specific changes in the ALs may also be a consequence of different exposures to the complex olfactory environment of foragers outside the colony (72).

The unique family of MRJPs is central for the RJB phenotype (73) and was therefore specifically investigated. However, even without a prior, MRJP2 warrants a special discussion because it is among the most up-regulated proteins in newly emerged and nurse RJB, but not foragers. The selection for RJ production in the RJBs may have increased the synthesis of the MRJPs in general and therefore the differences of MJRP abundance in the MBs and ALs might just be a passive consequence of an increased glandular MJRP production. However, our data showed differences for specific MRJPs and most of those differences only in the ALs and not the MBs. Together with the increasingly recognized diversity of biological functions of MRJPs described below, these findings could therefore indicate additional function of the MRJPs in the brain that contributes to the RJ syndrome. In addition to their classic functions in RJ as larval food source (74, 75, 76), MRJPs are important in the honeybee brain (77, 78), might be correlated with the learning ability of worker bees (79), and can affect Kenyon cells plasticity in the MBs (80). The highly abundant MRJP1–4 in the ALs of newly emerged RJBs, compared with ITBs, may increase the glomeruli plasticity and energy supply of the ALs. The increased levels of MRJP2 in RJBs compared with ITBs extend to the MBs and ALs in newly emerged and nurse bees. No relevant functions of MRJP2 in the honeybee brain have been identified yet, but it is expressed and up-regulated in WT compared with reproductively active workers (78). Hence, it is convincible that MRJP2 is important for the behavioral control of the normal nursing activities of workers by either regulatory functions or serving a nutritive function for neurons, which is also conceivable for other MRJPs.

## CONCLUSIONS

Our study determines the social phenotype, behavior and the proteome of the two important brain regions of two bee strains with distinct reproductive investment in the form of rearing the larvae in queen cells into queen bees. The distinct reproductive investment via alloparental care between both bee stocks is only marginally influenced by larval identity but is predominantly determined by the sensitivity of the olfactory response to larval pheromone. The subdivided comparison of the brain proteome of worker bees at three time points characterized the differences that might enable the RJB phenotype at the neurological level. Prominent differences in the protein profiles of the brain, particularly the antennal lobes, existed in newly emerged and nurse bees that were not found in foraging bees, despite numerous other proteome differences at the foraging stage. The overall results indicate divergent trajectories of brain development between the RJBs and ITBs based apparently on energy provision and metabolism. When the relevant nursing behavior is performed, differences also manifest in enhanced signal transduction, predominantly in the ALs, of the RJBs. In addition, our study supports findings that the MRJPs have been co-opted to serve important novel functions in the central nervous system. These results lay the foundation for further functional studies to manipulate the expression of the identified candidate proteins and investigate their specific neurobiological functions in regulating the complex social phenotype of reproductive investment in honeybees.

## DATA AVAILABILITY

The MS proteomics data that support the findings of this study, and annotated spectra of the proteins identified on the basis of single peptide are openly available in the ProteomeXchange Consortium via the iProX partner repository, data set identifier PXD014207.

The Agricultural Sciences and Technology Innovation Program (CAAS-ASTIP-2015-IAR) to Jianke Li
The earmarked fund for Modern Agro-Industry Technology Research System in China (CARS-44) to Jianke Li
The Beijing Municipal Natural Science Foundation (5182031) to Chuan Ma
