# Supplementary material for: The Neuroproteomic Basis of Enhanced Perception and Processing of Brood Signals That Trigger Increased Reproductive Investment in Honeybee (Apis mellifera) Workers
Source: Mol Cell Proteomics. 2020 Nov 25;19(10):1632–48. doi: 10.1074/mcp.RA120.002123 (PMC8014994; doi:10.1074/mcp.RA120.002123)
Supplement: Supplementary file 1 [file mmc1.zip › 160718_2_supp_556832_qcpjc8.pdf]

Supplemental Figure Legends

Fig. S1 The matrix performed the correlation coefficient between label-free quantification intensities in triplicates of proteome data. The color code follows the indicated values of correlation coefficient. (Pearson correlation coefficients were 0.98-0.99 among replicates of the same samples, ranged from 0.82-0.92 among different samples.

Fig. S2 Quality assessment of proteome data by label-free quantification. A, Hierarchical clustering analysis of differentially expressed proteins in each sample. B, Principal component analysis (PCA). The proteome of all the mushroom bodies (MBs) and antennal lobes (ALs) of unselected Italian bees (ITBs) and high royal jelly producing bees (RJBs), their differentiation measured in triplicates based on component 1 and component 2, which account for 31% and 21.1% of variability, respectively. (NEB: newly emerged bees, NB: nurse bees, FB: forager bees).

Fig. S3 The enriched functional classes and pathways of quantitative comparison by up-regulated proteins in the antennal lobes (ALs) of newly emerged bees (NEBs) of unselected Italian bees (ITBs) relative to royal jelly bees (RJBs) (S0=0.1, FDR=0.05). The percentage of genes/term represents the proportion of genes enriched in the respective functional group. Identical color summarizes bars from the same functional group. For details of the enrichment analysis results, see Table S4. "*" represents $p<0.05$; "**" represents $p<0.01$.

Fig. S4 Venn diagram showing the shared and unique protein groups identified. A. Venn diagram showing the shared and unique protein groups identified in the mushroom bodies (MBs) of nurse bees (NB) between unselected Italian (ITBs) and high royal jelly producing (RJBs) honeybee stocks (>98% are shared). B. Venn diagram showing the shared and unique protein groups identified in the ALs of foragers bees (FB) between ITBs and RJBs (>95% are shared). C. Venn diagram showing the shared and unique protein groups identified in the MBs of FB between ITBs and RJBs (>98% are shared).

Fig. S5 The enriched functional classes and pathways of quantitative comparison by up-regulated proteins in the antennal lobes (ALs) of nurse bees (NBs) of unselected Italian bees (ITBs) relative to high royal jelly producing bees (RJBs) (S0=0.1, FDR=0.05). The percentage of genes/term represents the proportion of genes enriched in the respective functional group. Identical color summarizes bars from the same functional group. For details of the enrichment analysis results, see Table S10. "*" represents $p<0.05$; "**" represents $p<0.01$.

Fig. S6 The enriched functional classes and pathways of quantitative comparison by up-regulated proteins in the mushroom bodies (MBs) of nurse bees of high royal jelly producing bees (RJBs) relative to unselected Italian bees (ITBs) (S0=0.1, FDR=0.05). The percentage of genes/term represents the proportion of genes enriched in the respective functional group. For details of the enrichment analysis results, see Table S13. "*" represents $p<0.05$.

Fig. S7 The enriched functional classes and pathways of quantitative comparison by up-regulated proteins in the antennal lobes (ALs) of forager bees between high royal jelly producing bees (RJBs) relative to unselected Italian bees (ITBs) (S0=0.2, FDR=0.05). The percentage of genes/term represents the proportion of genes enriched in the respective functional group. Identical color summarizes bars from the same functional group. For details of the enrichment analysis results, see Table S16. "*" represents $p<0.05$; "**" represents $p<0.01$.

Fig. S1

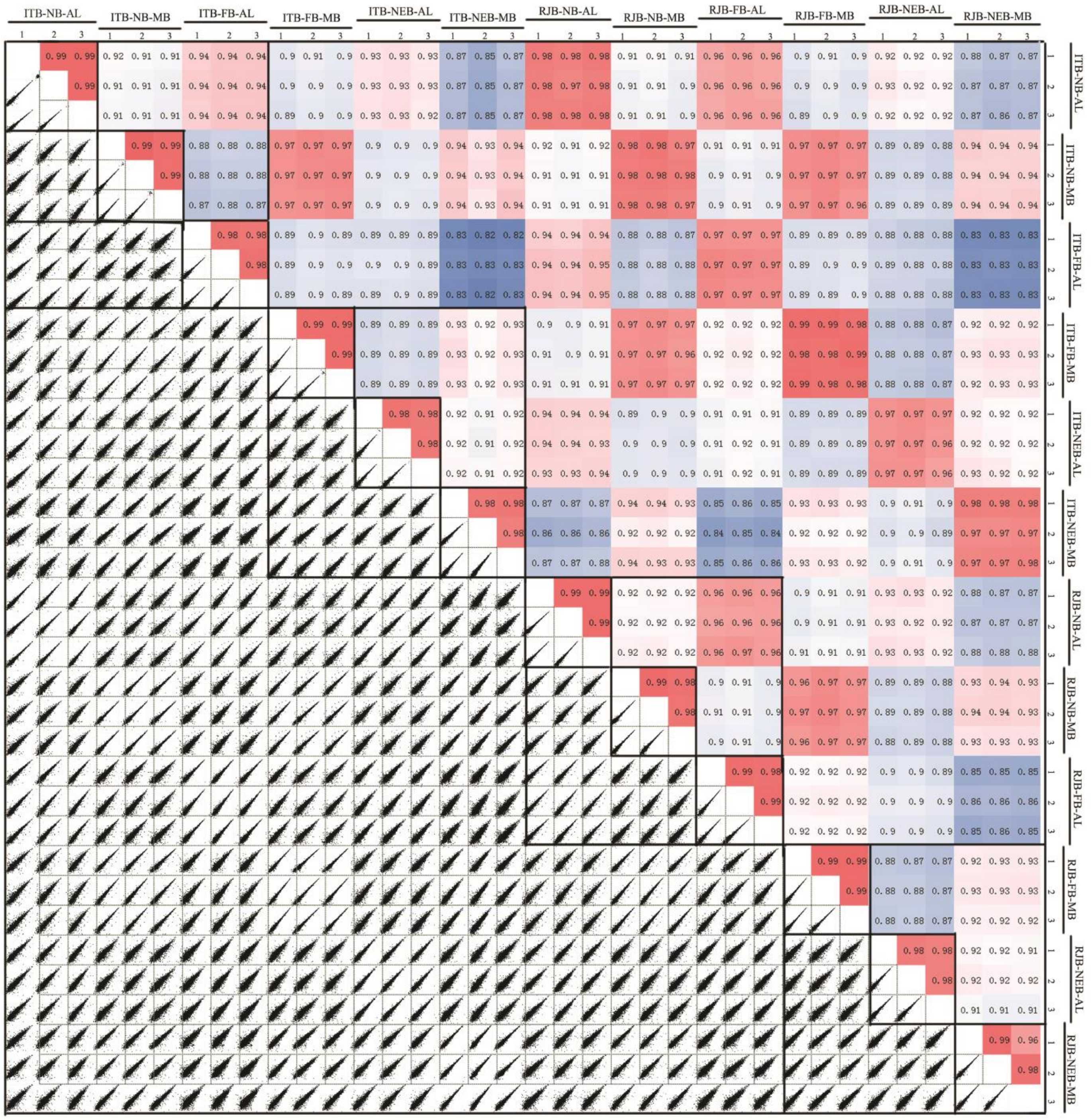
ITB-NB-AL
ITB-NB-MB
ITB-FB-AL
ITB-FB-MB
ITB-NEB-AL
ITB-NEB-MB
RJB-NB-AL
RJB-NB-MB
RJB-FB-AL
RJB-FB-MB
RJB-NEB-AL
RJB-NEB-MB

Fig. S2

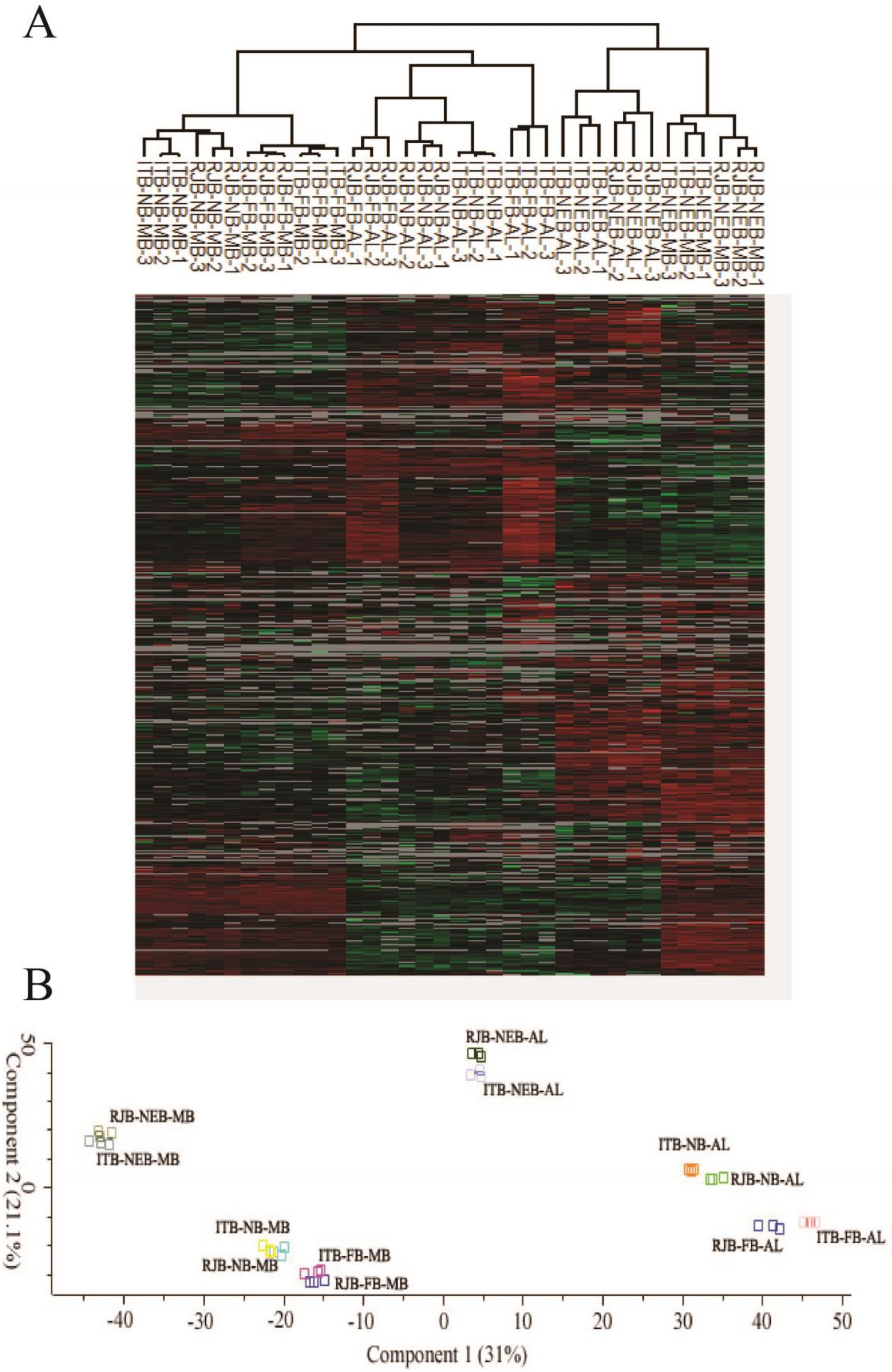
A
ITB-NB-MB-3
ITB-NB-MB-2
ITB-NB-MB-1
RJB-NB-MB-3
RJB-NB-MB-2
RJB-NB-MB-1
RJB-FB-MB-2
RJB-FB-MB-3
RJB-FB-MB-1
ITB-FB-MB-2
ITB-FB-MB-1
ITB-FB-MB-3
RJB-FB-AL-1
RJB-FB-AL-2
RJB-FB-AL-3
RJB-NB-AL-2
RJB-NB-AL-3
RJB-NB-AL-1
ITB-NB-AL-3
ITB-NB-AL-2
ITB-NB-AL-1
ITB-FB-AL-1
ITB-FB-AL-2
ITB-FB-AL-3
ITB-NEB-AL-3
ITB-NEB-AL-2
ITB-NEB-AL-1
RJB-NEB-AL-2
RJB-NEB-AL-1
RJB-NEB-AL-3
ITB-NEB-MB-3
ITB-NEB-MB-2
ITB-NEB-MB-1
RJB-NEB-MB-3
RJB-NEB-MB-2
RJB-NEB-MB-1
B
RJB-NEB-AL
ITB-NEB-AL
RJB-NEB-MB
ITB-NEB-MB
ITB-NB-AL
RJB-NB-AL
RJB-FB-AL
ITB-FB-AL
ITB-NB-MB
RJB-NB-MB
ITB-FB-MB
RJB-FB-MB
Component 2 (21.1%)
50
0
-40
-30
-20
-10
0
10
20
30
40
50
Component 1 (31%)

Fig. S3

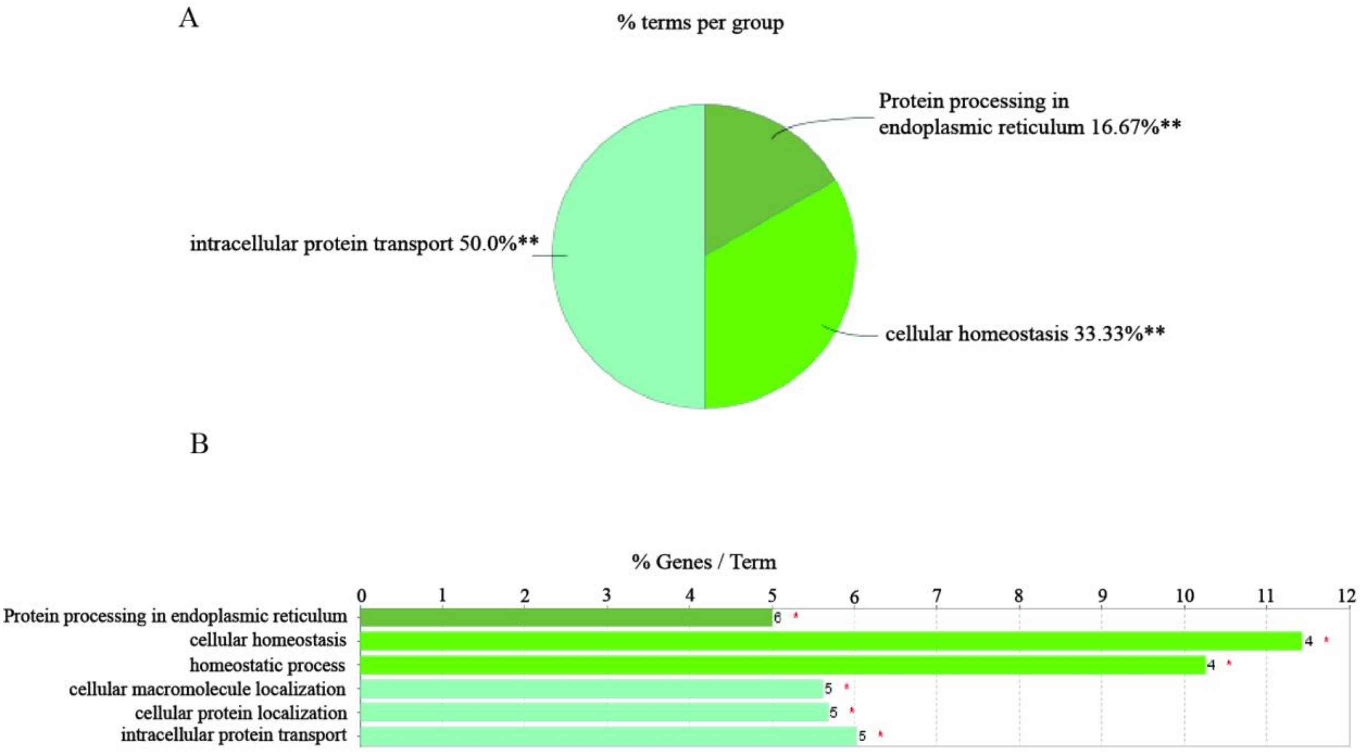
A
% terms per group
Protein processing in endoplasmic reticulum 16.67%**
intracellular protein transport 50.0%**
cellular homeostasis 33.33%**
B
% Genes / Term
0 1 2 3 4 5 6 7 8 9 10 11 12
Protein processing in endoplasmic reticulum 6 *
cellular homeostasis 4 *
homeostatic process 4 *
cellular macromolecule localization 5 *
cellular protein localization 5 *
intracellular protein transport 5 *

Fig. S4

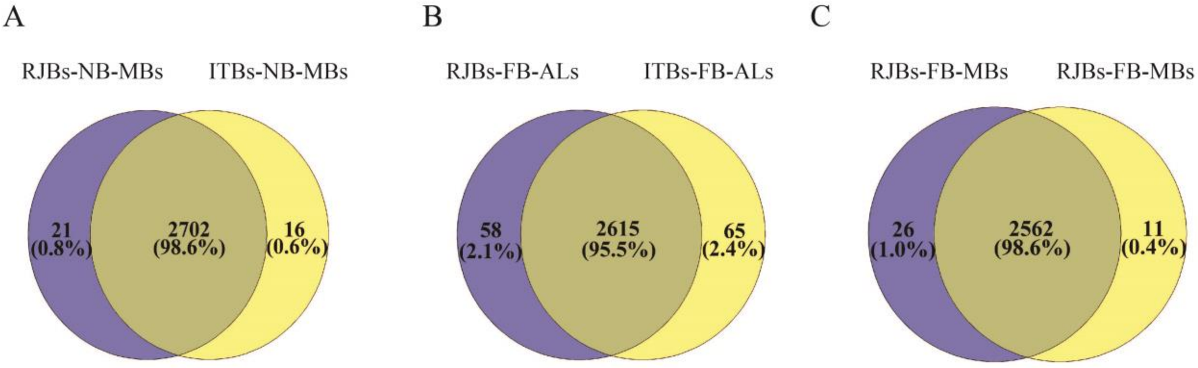
A
RJBs-NB-MBs
ITBs-NB-MBs
21 (0.8%)
2702 (98.6%)
16 (0.6%)
B
RJBs-FB-ALs
ITBs-FB-ALs
58 (2.1%)
2615 (95.5%)
65 (2.4%)
C
RJBs-FB-MBs
RJBs-FB-MBs
26 (1.0%)
2562 (98.6%)
11 (0.4%)

Fig. S5

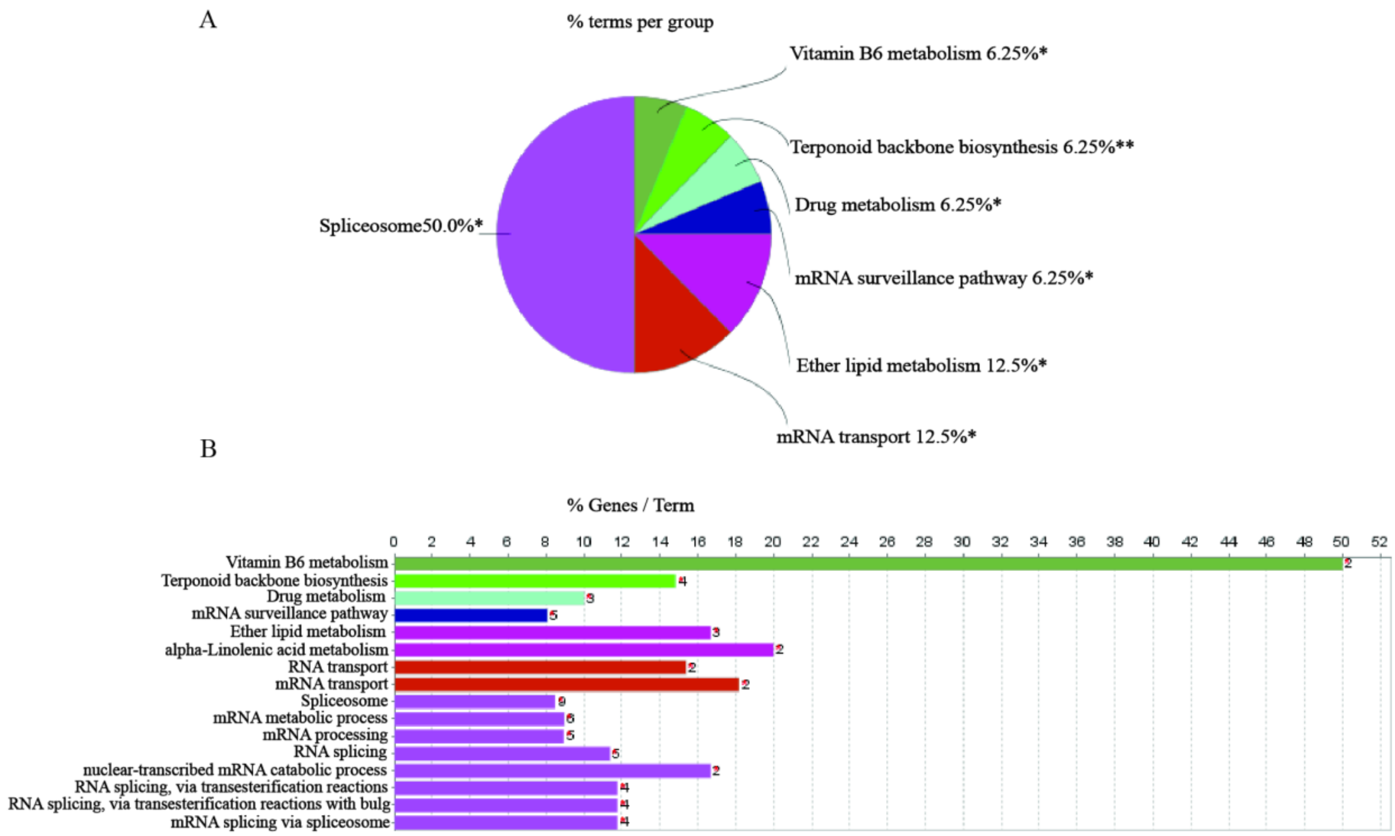
A
% terms per group
Vitamin B6 metabolism 6.25%*
Terponoid backbone biosynthesis 6.25%**
Drug metabolism 6.25%*
mRNA surveillance pathway 6.25%*
Ether lipid metabolism 12.5%*
mRNA transport 12.5%*
Spliceosome50.0%*
B
% Genes / Term
0 2 4 6 8 10 12 14 16 18 20 22 24 26 28 30 32 34 36 38 40 42 44 46 48 50 52
Vitamin B6 metabolism 2
Terponoid backbone biosynthesis 4
Drug metabolism 3
mRNA surveillance pathway 5
Ether lipid metabolism 3
alpha-Linolenic acid metabolism 2
RNA transport 2
mRNA transport 2
Spliceosome 9
mRNA metabolic process 6
mRNA processing 5
RNA splicing 5
nuclear-transcribed mRNA catabolic process 2
RNA splicing, via transesterification reactions 4
RNA splicing, via transesterification reactions with bulg 4
mRNA splicing via spliceosome 4

Fig. S6

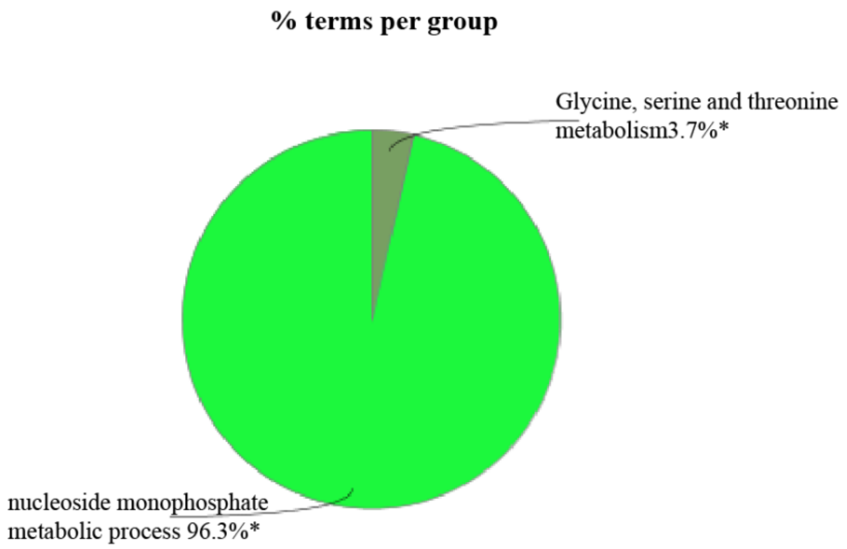
% terms per group
Glycine, serine and threonine metabolism3.7%*
nucleoside monophosphate metabolic process 96.3%*

Fig. S7

A

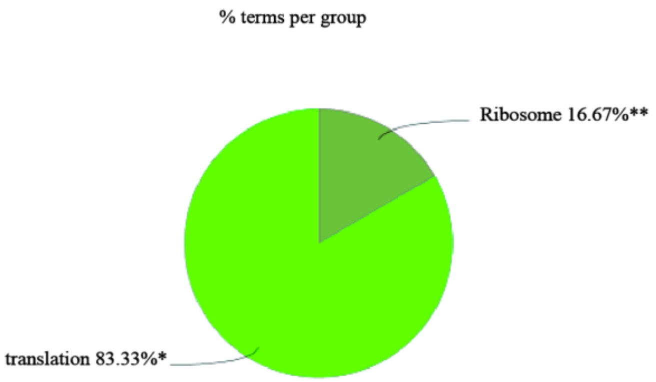
% terms per group
Ribosome 16.67%**
translation 83.33%*

B

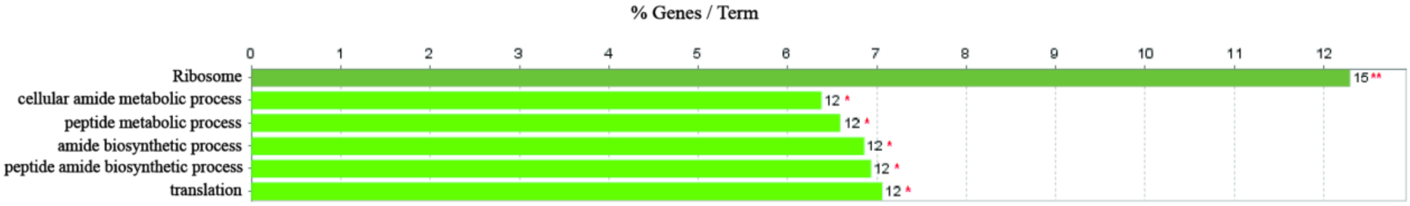
% Genes / Term
0
1
2
3
4
5
6
7
8
9
10
11
12
Ribosome
cellular amide metabolic process
peptide metabolic process
amide biosynthetic process
peptide amide biosynthetic process
translation
15**
12 *
12 *
12 *
12 *
12 *
