# Supplementary material for: The Neuroproteomic Basis of Enhanced Perception and Processing of Brood Signals That Trigger Increased Reproductive Investment in Honeybee (Apis mellifera) Workers
Source: Mol Cell Proteomics. 2020 Nov 25;19(10):1632–48. doi: 10.1074/mcp.RA120.002123 (PMC8014994; doi:10.1074/mcp.RA120.002123)
Supplement: Supplementary file 1 [file mmc1.zip › 160718_2_supp_561966_qdfzrs.pdf]

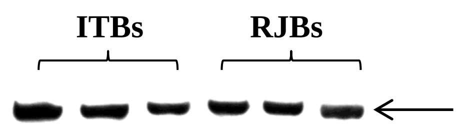

WB-Original image 1: NB-AL-β actin (Molecular weight: 42 kDa) monoclonal antibody

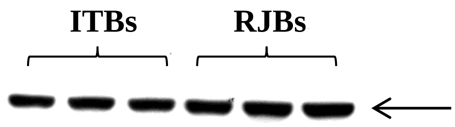

WB-Original image 2: NB-AL-MRJP2 (Molecular weight: 51 kDa) monoclonal antibody

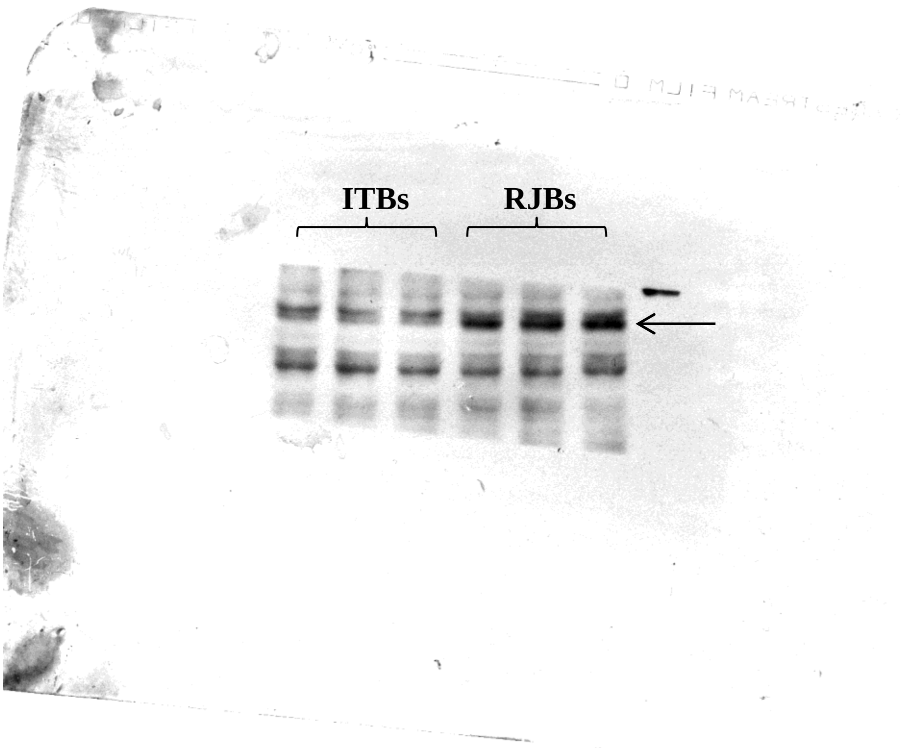

WB-Original image 3: NEB-AL-MRJP2 (51 kDa) monoclonal antibody

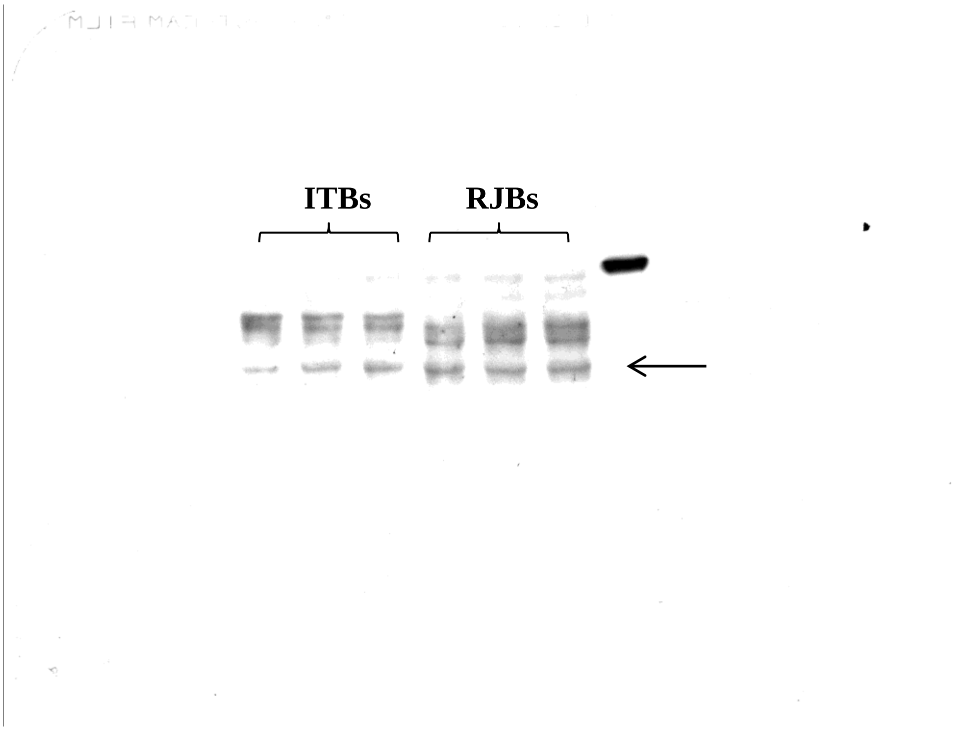

WB-Original image 4: NEB-MB-MRJP2 (Molecular weight: 51 kDa) monoclonal antibody

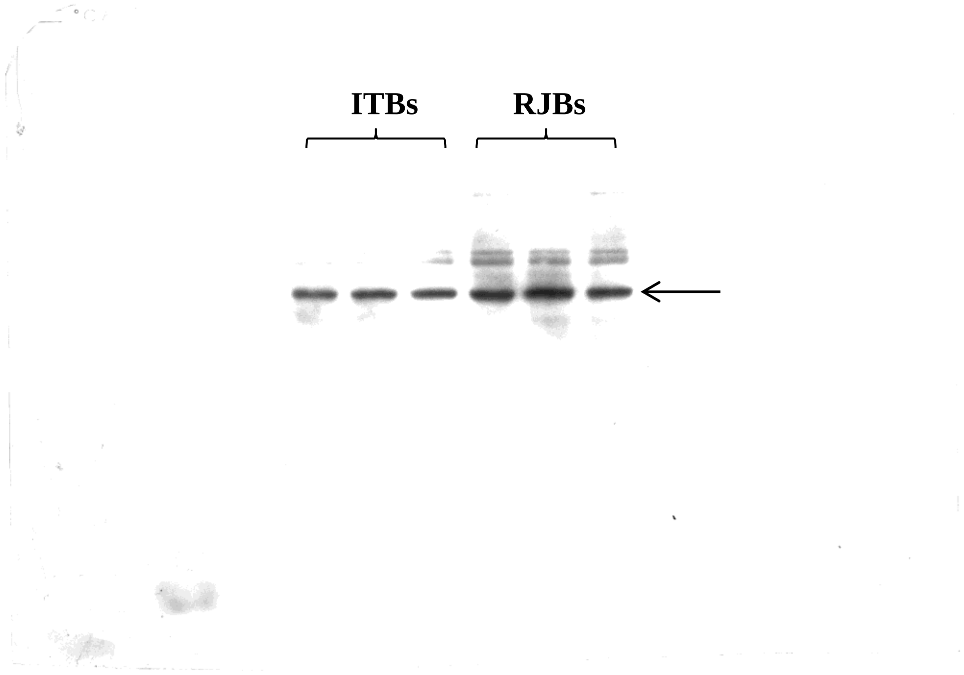

WB-Original image 5: NB-AL-PKG (Molecular weight: 74.5 kDa) polyclonal antibody

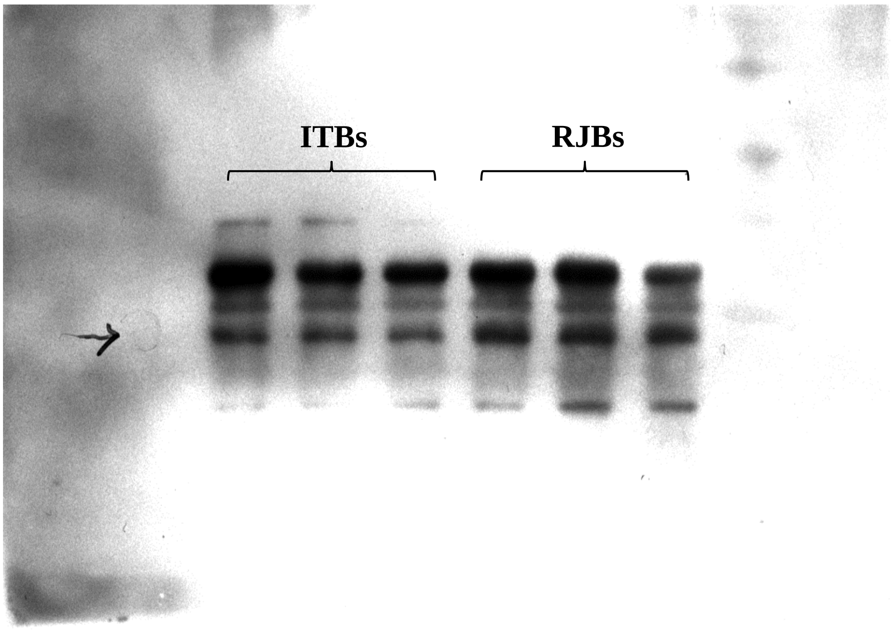

WB-Original image 6: NB-AL-Syx17 (Molecular weight: 33 kDa) polyclonal antibody

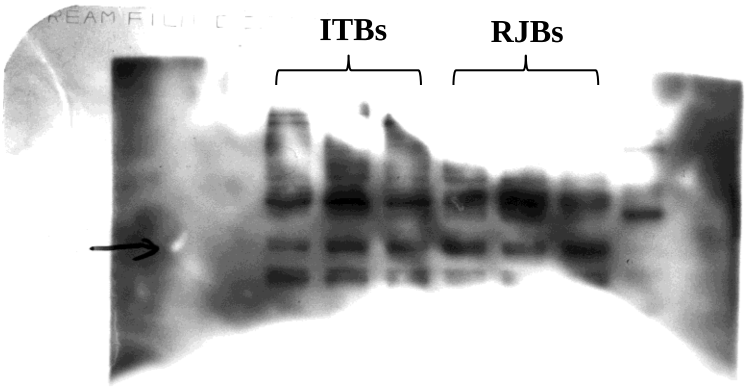

WB-Original image 7: NB-AL-Src64B (Molecular weight: 57.3 kDa) polyclonal antibody

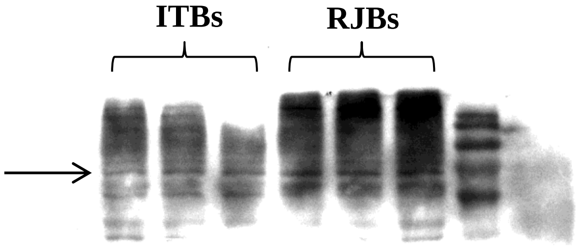

WB-Original image 8: NB-AL-PAK3 (Molecular weight: 64 kDa) polyclonal antibody

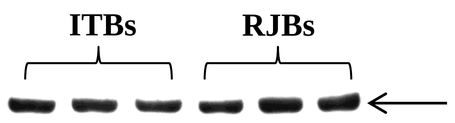

WB-Original image 9: NEB-AL-β-actin (Molecular weight: 42 kDa) monoclonal antibody

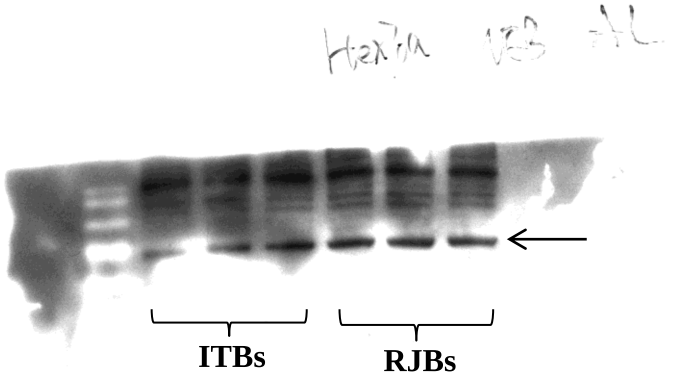

WB-Original image 10: NEB-AL-Hex70a (Molecular weight: 75 kDa) polyclonal antibody

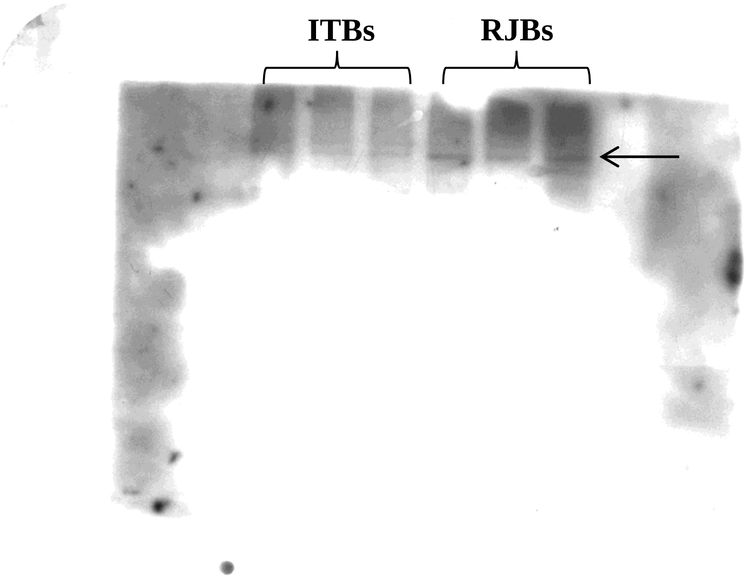

WB-Original image 11: NEB-AL-Hex70b (Molecular weight: 75 kDa) polyclonal antibody

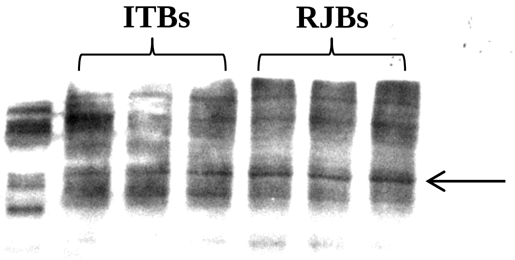

WB-Original image 12: NEB-AL-Hex70C (Molecular weight: 75 kDa) polyclonal antibody

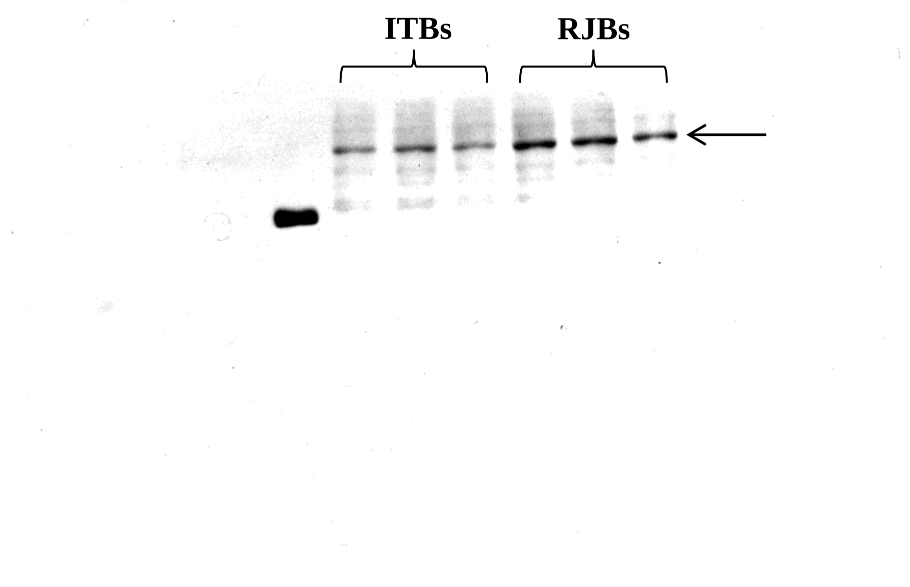

WB-Original image 13: NEB-AL-Hex110 (Molecular weight: 112 kDa) monoclonal antibody

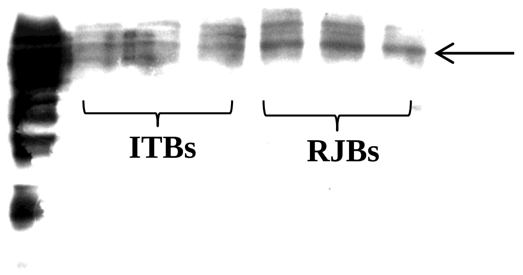

WB-Original image 14: NEB-AL-Vg (Molecular weight: 192 kDa) polyclonal antibody

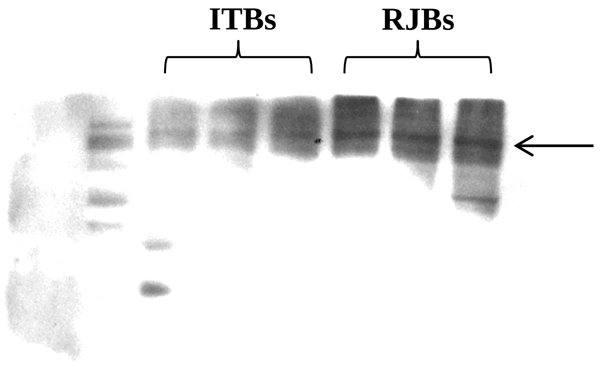

WB-Original image 15: NEB-AL-VHDL (Molecular weight: 165 kDa) polyclonal antibody

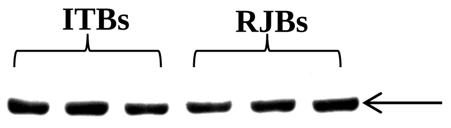

WB-Original image 16: NEB-AL- β-actin (Molecular weight: 42 kDa) monoclonal antibody

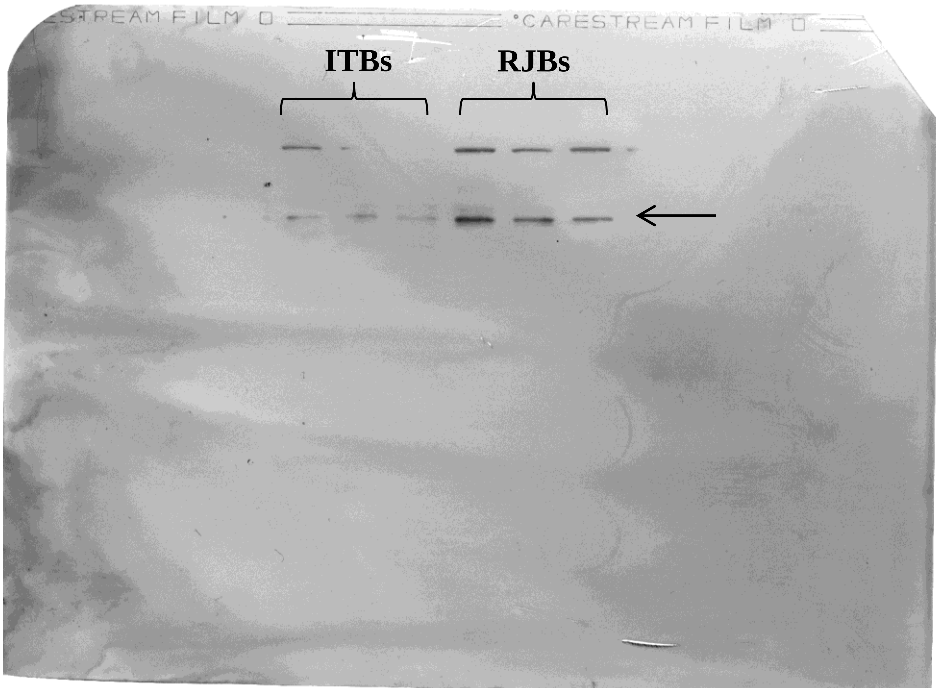

WB-Original image 17: NEB-AL-MRJP1 (Molecular weight: 60 kDa) monoclonal antibody

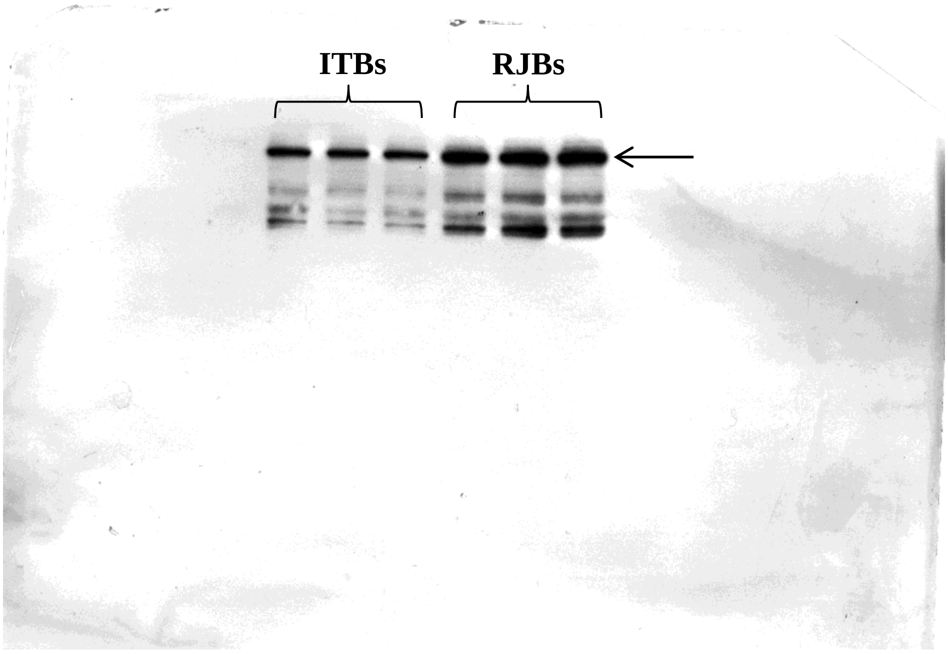

WB-Original image 18: NEB-AL-MRJP3 (Molecular weight: 60-70 kDa) monoclonal antibody

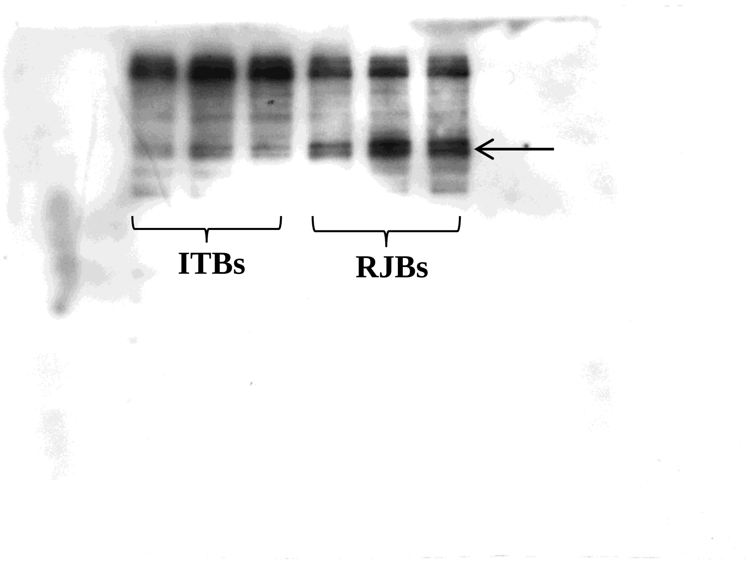

WB-Original image 19: NEB-AL-MRJP4 (Molecular weight: 53 kDa) monoclonal antibody

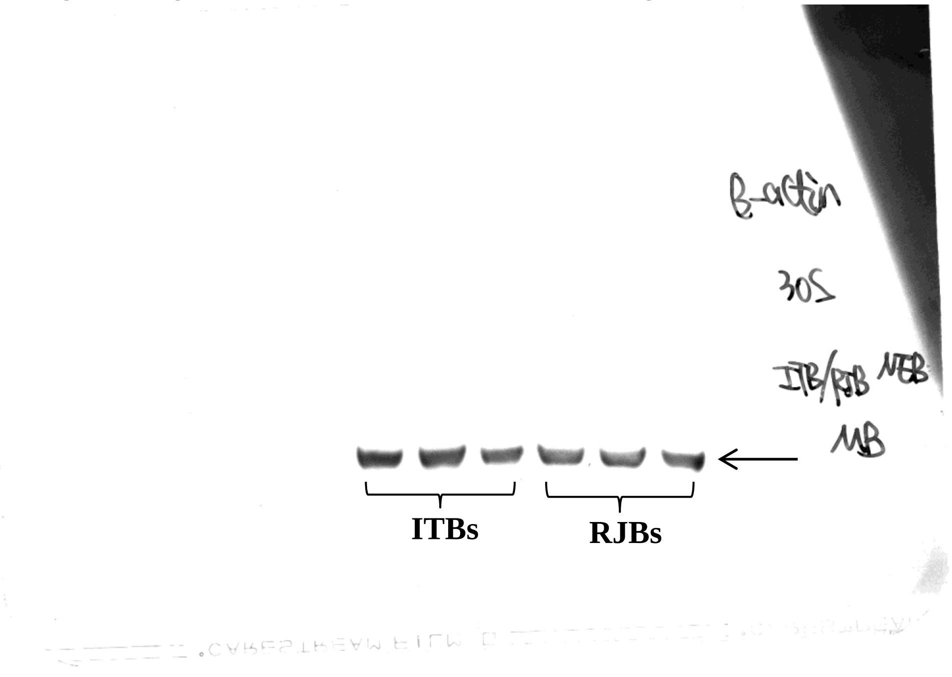

WB-Original image 20: NEB-MB- β-actin (Molecular weight: 42 kDa) monoclonal antibody

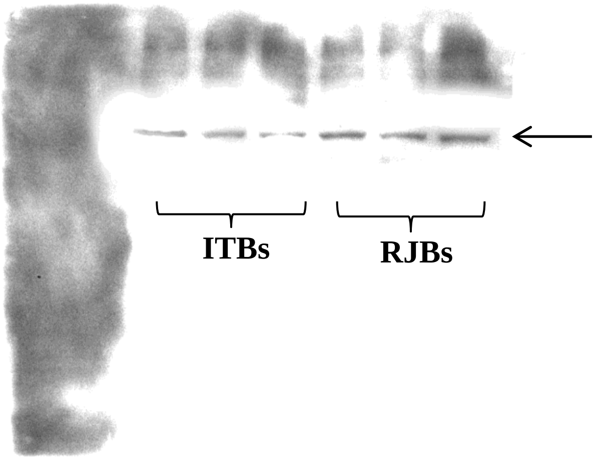

WB-Original image 21: NEB-MB-Hex70a (Molecular weight: 75 kDa) polyclonal antibody

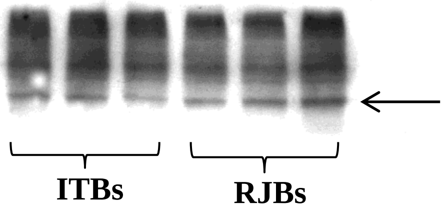

WB-Original image 22: NEB-MB-Hex70B (Molecular weight: 75 kDa) polyclonal antibody

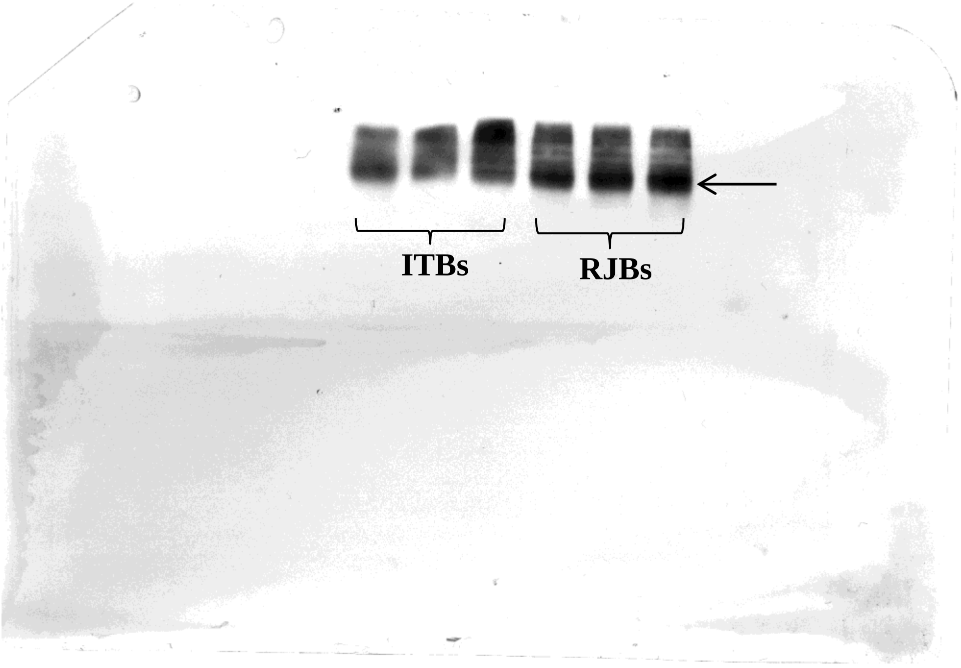

WB-Original image 23: NEB-MB-Hex70C (Molecular weight: 75 kDa) polyclonal antibody

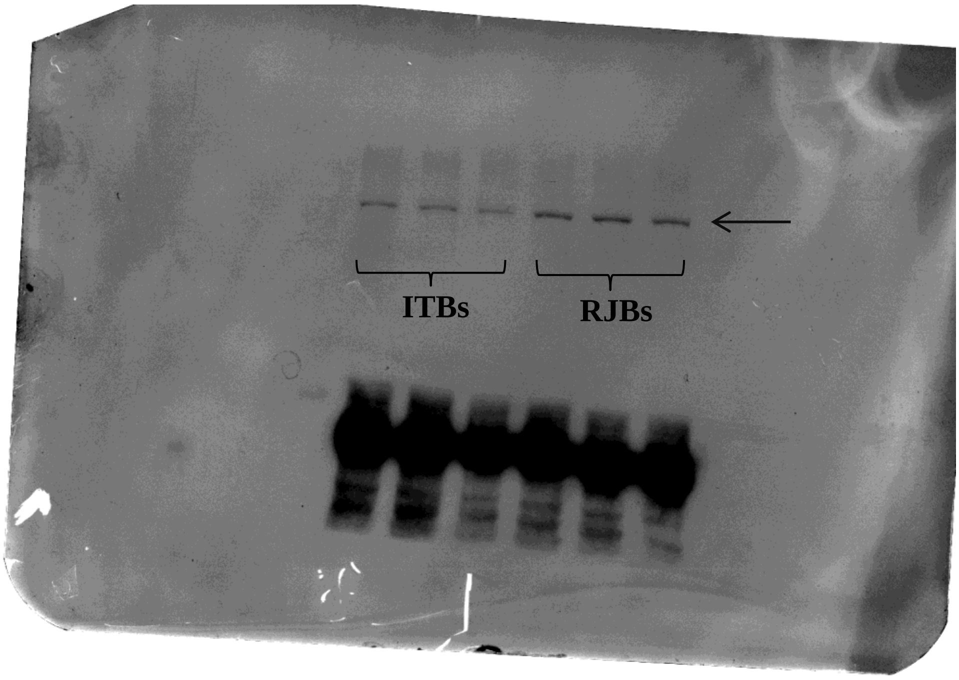

WB-Original image 24: NEB-MB-Hex110 (Molecular weight: 112 kDa) monoclonal antibody

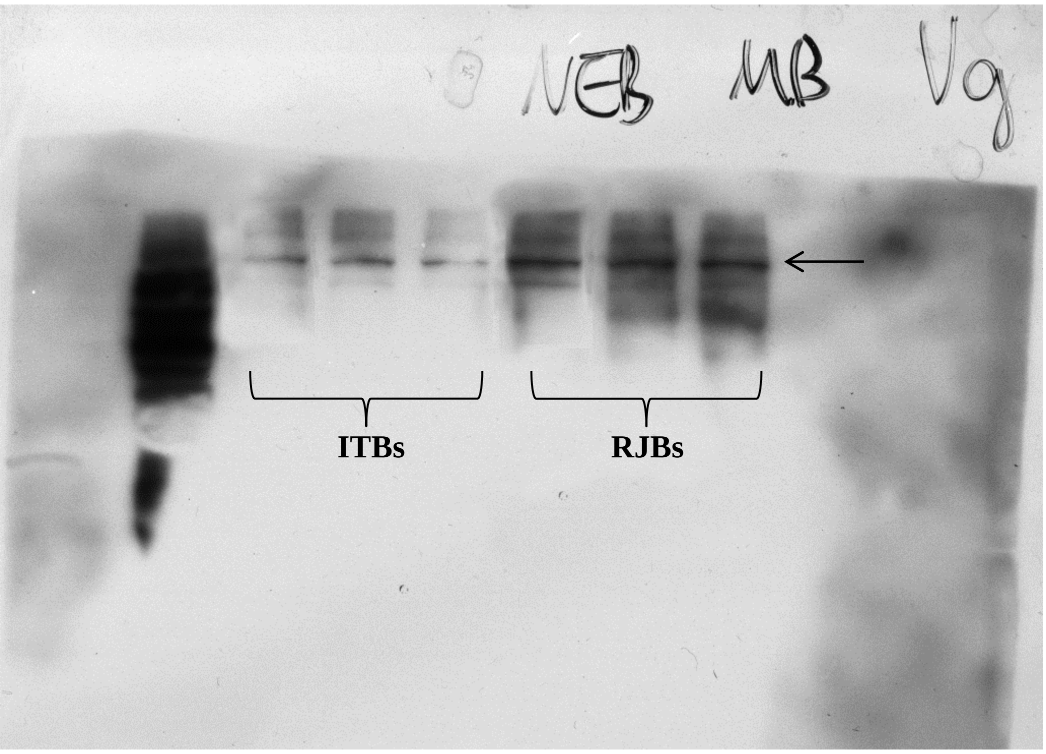

WB-Original image 25: NEB-MB-Vg (Molecular weight: 192 kDa) polyclonal antibody

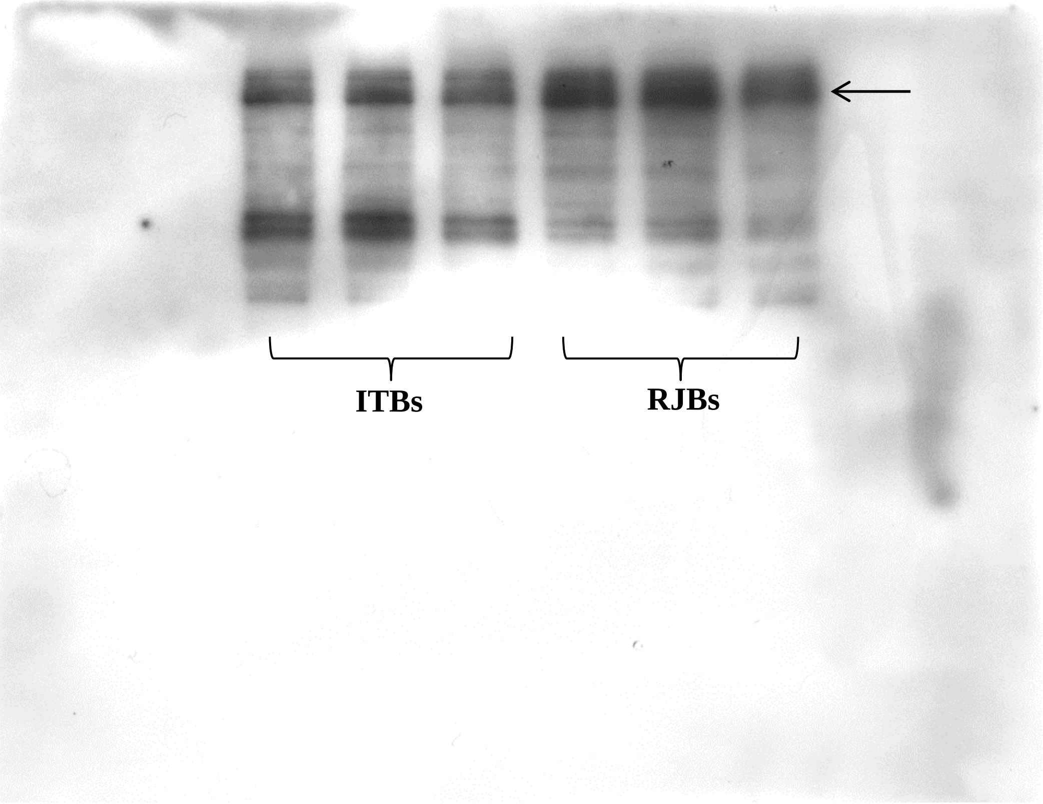

WB-Original image 26: NEB-MB-VHDL (Molecular weight: 165 kDa) polyclonal antibody
